# Fast operando spectroscopy tracking in situ generation of rich defects in silver nanocrystals for highly selective electrochemical CO_2_ reduction

**DOI:** 10.1038/s41467-021-20960-8

**Published:** 2021-01-28

**Authors:** Xinhao Wu, Yanan Guo, Zengsen Sun, Fenghua Xie, Daqin Guan, Jie Dai, Fengjiao Yu, Zhiwei Hu, Yu-Cheng Huang, Chih-Wen Pao, Jeng-Lung Chen, Wei Zhou, Zongping Shao

**Affiliations:** 1 State Key Laboratory of Materials-Oriented Chemical Engineering, College of Chemical Engineering, Nanjing Tech University, Nanjing, P. R. China; 2 Max Planck Institute for Chemical Physics of Solids, Dresden, Germany; 3 Department of Electrophysics, National Chiao Tung University, Hsinchu, Taiwan; 4 National Synchrotron Radiation Research Center, Hsinchu, Taiwan; 5 Department of Chemical Engineering, Curtin University, Perth, WA Australia

**Keywords:** Electrocatalysis, Carbon capture and storage, Electrocatalysis

## Abstract

Electrochemical CO_2_ reduction (ECR) is highly attractive to curb global warming. The knowledge on the evolution of catalysts and identification of active sites during the reaction is important, but still limited. Here, we report an efficient catalyst (Ag-D) with suitable defect concentration operando formed during ECR within several minutes. Utilizing the powerful fast operando X-ray absorption spectroscopy, the evolving electronic and crystal structures are unraveled under ECR condition. The catalyst exhibits a ~100% faradaic efficiency and negligible performance degradation over a 120-hour test at a moderate overpotential of 0.7 V in an H-cell reactor and a current density of ~180 mA cm^−2^ at −1.0 V vs. reversible hydrogen electrode in a flow-cell reactor. Density functional theory calculations indicate that the adsorption of intermediate COOH could be enhanced and the free energy of the reaction pathways could be optimized by an appropriate defect concentration, rationalizing the experimental observation.

## Abstract (web summary)

Efficient electrocatalysts are crucial to the electrochemical carbon dioxide reduction. Here, the authors use the fast operando technique to explore a silver-based catalyst with improved catalytic performance benefiting from the suitable defect structures.

## Introduction

The extensive consumption of coal, oil and other fossil fuels has resulted in the emission of enormous amounts of carbon dioxide (CO_2_) into the surrounding atmosphere, which is the culprit of the global greenhouse effect^1–4^. Therefore, the management of CO_2_ concentrations in the atmosphere has become an important topic^5^. The use of clean carbon-neutral fuel systems, CO_2_ sequestration, and CO_2_ transformation into high value^−^added products are several strategies for inhibiting continuous increases in or even reducing the CO_2_ concentration in the atmosphere^6^. Among these techniques, electrochemical CO_2_ reduction (ECR) driven by clean and renewable electricity sources (e.g. solar energy) is particularly promising^7,8^, and can synthesize a wide variety of chemicals, such as formic acid, carbon monoxide (CO), alcohol and methane, along with the elimination of CO_2_^9–13^. Among the synthesized chemicals, CO is regarded as having the most potential to achieve positive gross margins in terms of techno-economic assessments^6,14,15^. However, due to the small difference in standard reaction potentials for different products, it is a substantial challenge to achieve high selectivity towards one specific chemical, which requires the adoption of highly selective and efficient electrocatalysts^16^.

Among various electrocatalysts for ECR to CO investigated over past decades, metal-based materials^11^, especially metallic Ag^17,18^, may be the most promising candidate for industrial development in view of their remarkable performance and economic viability compared to noble Au-based materials^19^. It has been found that the performance (selectivity, activity and stability) of Ag-based catalysts is strongly related to their morphologies, shapes, particle sizes, crystal facets, electronic structures and so on^17^. Unmodified polycrystalline Ag metal, such as traditional Ag foil, shows an unsatisfactory CO faradaic efficiency (FE) of only 70–80%. Various design strategies for novel Ag catalysts^17^, such as shape controlling^20^, alloying^21^ and the construction of nanostructures^22,23^, have therefore been proposed to optimize the performance of ECR to CO. In particular, defect engineering has been identified as a significant method to enhance reactivity^3,24^. For instance, it has been proposed that plasma oxidation pretreatment of Ag foil could introduce an enhanced content of low-coordinated active sites, leading to an improved FE and a remarkably reduced overpotential (*η*) for ECR to CO^25^. Electrochemical reduction of metal oxides, such as Au oxides and Cu oxides, could also introduce vacancies or other defects to result in better performance^26,27^; this approach is highly attractive because of its simplicity compared to plasma treatment. However, how electrochemical reduction facilitates defect generation is still unclear.

Advanced operando X-ray, optical and electron-based characterizations may provide useful information about defect generation under real reaction conditions^28–31^. Among these methods, X-ray absorption spectroscopy (XAS) probes atom-specific structural details of catalysts^12,32,33^. X-ray absorption near edge structure (XANES) concentrates on the characteristic information within ±1% of the interested edge, uncovering the electronic configuration of the absorber atom^28,32^. Meanwhile, extended X-ray absorption fine structure (EXAFS) spectra offer clues on the local coordinated environments from their oscillating intensity^28,34,35^. Although the operando XAS technique has exhibited advantages in studying catalysts for some electrocatalytic reactions, such as the oxygen evolution reaction^36,37^ and hydrogen evolution reaction (HER)^38^, relevant reports on ECR are still limited. In addition, considering the fast transition process of catalysts during electrochemical reactions, which usually finish within several minutes^39^, the conventional XAS technique hardly meets the needs of operando investigation. The conventional XAS may take tens of minutes to obtain a single spectrum, thus giving rise to an incorrect information on the crystal and electronic structure, namely an average of many different states of the evolving catalysts, and the reliability depending on the evolving times from materials to materials. Fast XAS technique with excellent time^−^resolution should be pursued to understand the mechanism and the formation process of active sites in catalysts during the electrochemical reaction, which is vital for the rational design of new catalysts^36,40,41^.

In terms of the theoretical study, the density functional theory (DFT) calculations have playing an important role in the mechanistic investigations and the theory-guided catalyst design. For instance, Jens K. Nørskov et al. investigated the activity descriptors for ECR to methane based on an extensive set of DFT calculations^42^. Recently, the landmark achievement has been made with the help of the DFT calculations and active machine learning, which accelerates the discovery of catalysts for ECR^43^. The mechanistic insights into ECR over the Ag electrode has also been reported, benefiting from the DFT calculations^2,20,25^. Inspiring from these results, the role of the defect engineering in the enhancement of the ECR performance may be explored by DFT calculations.

Herein, we apply a fast operando XAS technique to track the time-dependent composition and structure evolution of Ag_2_O precatalyst for ECR, thus to obtain useful information to understand the structure-performance relationship. The Ag–O bond is found to be quickly broken by electrons from the cathode, resulting in the formation of a nanostructured silver catalyst. We also observe that massive defect structures are effectively endowed to the Ag catalyst during operando electrochemical reduction. Excitingly, the Ag-D catalyst exhibits a nearly 100% CO_2_-to-CO FE under a broad range of potentials (−0.8 to −1.0 V vs. reversible hydrogen electrode (RHE)) and maintains nearly unchanged CO selectivity over a more than 120-hour constant-potential test, which is superior to most reported Ag-based electrocatalysts. To optimize the mass transport of CO_2_ for a higher current density towards industry-level application^44^, the Ag-D electrocatalyst is further fabricated into a gas diffusion electrode (GDE) and tested using a microfluidic flow reactor, which shows a total current density up to 180 mA cm^−2^ with a 92–100% CO FE at a moderate overpotential range. The experimental results and density functional theory (DFT) calculations conjointly reveal the critical role of defect structures in Ag-D catalysts for outstanding ECR performance.

## Results

### Synthesis and characterization of Ag_2_O and Ag-D

**Fig. 1:**
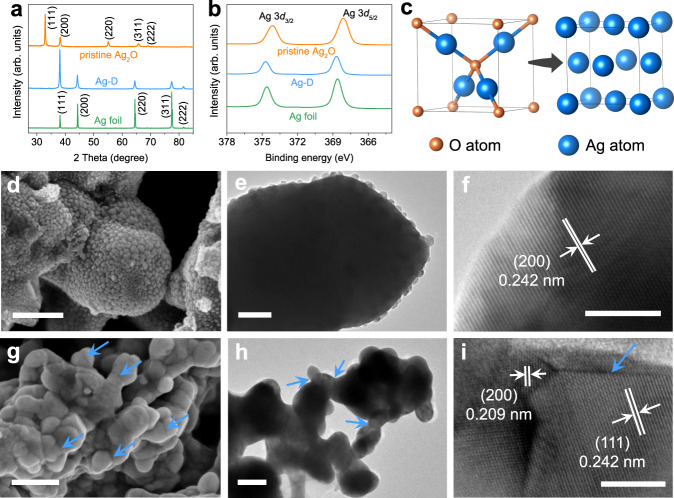
Materials characterization of Ag_2_O, Ag-D and Ag foil samples. **a** XRD patterns. **b** Ag 3*d* XPS spectra. **c** Crystal structure schematics of Ag_2_O (left) and Ag (right). **d, g** SEM images of Ag_2_O and Ag-D samples. **e, h** Typical TEM images of Ag_2_O and Ag-D samples. **f, i** HRTEM images of Ag_2_O and Ag-D samples. Scale bar in **d**, **g** is 250 nm, in **e** is 50 nm, in **h** is 100 nm and in **f**, **i** is 5 nm.

Ag_2_O as a catalyst precursor was prepared by a facile precipitation reaction of NaOH and AgNO_3_, followed by suction filtration and drying (Supplementary Fig. 1a). The powder X-ray diffraction (XRD) pattern exhibited obvious peaks at 2-theta values of 32.8°, 38.1°, 54.9° and 65.4°, as shown in Fig. 1a, corresponding to the (111), (200), (220) and (311) diffraction planes of the Ag_2_O phase (PDF:00-041-1104), respectively. The Ag oxidation states were measured by X-ray photoelectron spectroscopy (XPS). A lower binding energy of Ag 3*d*_5/2_ at ~368.1 eV in Ag_2_O compared to that of Ag 3*d*_5/2_ (~368.6 eV) in silver foil was observed (Fig. 1b), confirming Ag(I) species in Ag_2_O^39,45^. Moreover, the absence of any satellite structure indicated that no AgO has been formed^45^. The scanning electron microscopy (SEM) image, combined with the low-magnification transmission electron microscopy (TEM) image, confirmed a unique ‘sesame stick’-like particulate morphology structure (Fig. 1d and 1e). Abundant and uniform nanoparticles (~10–30 nm in diameter) were located on the surface of larger bulk particles. The special morphology of Ag_2_O may be one of the reasons for the formation of the nanostructured Ag-D catalyst, which will be further discussed later. The high-resolution transmission electron microscopy (HRTEM, Fig. 1f) revealed a lattice distance of 2.42 Å in the particle, which was indexed to the (200) plane of cubic Ag_2_O. The obtained Ag_2_O powder was mixed with Super P, ethanol and Nafion solution to make an electrode ink, which was subsequently dropped onto a glassy carbon electrode (GCE). During the initial stage of ECR process, the Ag_2_O was operando converted into metallic Ag (Ag-D) at the beginning of the reaction (Supplementary Fig. 1b). The clear change in electrode colour from dark brown to yellow over time is shown in Supplementary Fig. 2. No bubbles on the surface of the electrode could be observed by the naked eye until the transformation from Ag_2_O to Ag ended. The transformation process and final structure of the catalyst were further confirmed by ex situ characterization techniques. Time-dependent ex situ XPS revealed a positive shift in the binding energy of the Ag 3*d*_5/2_ peak, confirming the reduction of its valence state (Fig. 1b, Supplementary Fig. 3a). The SEM image of the catalyst after experiencing a 15-second electrochemical reduction time (Supplementary Fig. 3b) revealed two kinds of particles with different sizes, indicating the intermediate state of the transformation. The final crystal structure of cubic Ag-D was demonstrated from ex situ XRD and HRTEM. No characteristic XRD peaks corresponding to Ag_2_O were observed (Fig. 1a). The Ag-D sample showed expected spacings of 2.09 Å and 2.42 Å corresponding well to the (200) and (111) lattice planes (Fig. 1i), respectively. A schematic depicting the unit cell change from Ag_2_O crystal to Ag crystal is shown in Fig. 1c. Both the XRD and XPS results demonstrated the complete reduction of Ag_2_O to Ag. These results were basically consistent with previous studies for several oxide-derived metal catalysts^26,27,39^.

**Fig. 2:**
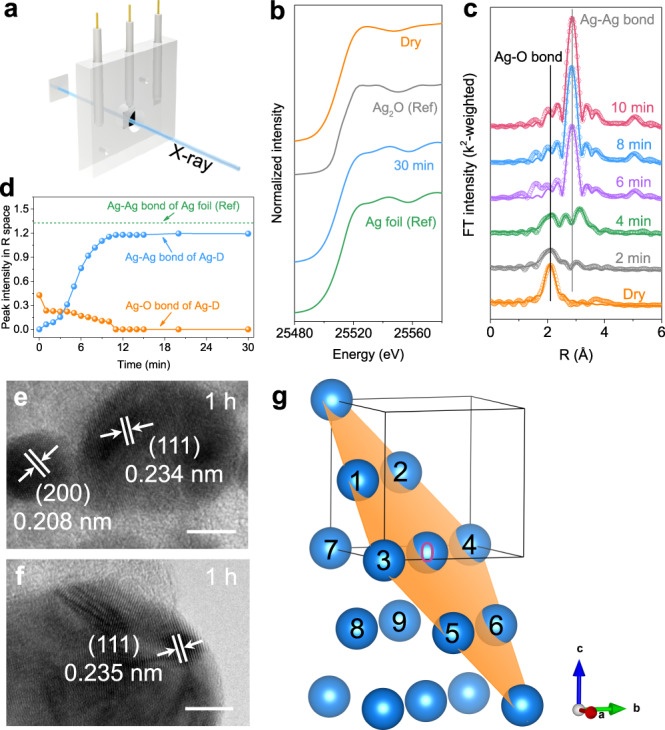
Fast operando XAS experiments. **a** Schematic illustration of the reactor for operando XAS. **b** Normalized XANES profiles. **c** Time-resolved k^2^-weighted FT EXAFS spectra (circles) and the fitting result (lines) for the Ag-D catalyst. **d** Time-resolved peak intensity of the Ag–Ag bond and Ag–O bond for the Ag-D catalyst. **e**, **f** HRTEM images at different positions of the Ag-D catalyst after a one-hour reaction. **g** Schematic illustration of the local environments of Ag atoms in the (111) plane. Scale bar in **e** and **f** is 5 nm.

Despite the similar crystal structure between Ag-D and the polycrystalline Ag foil, their morphologies were dramatically different, as revealed by electron microscopy. The pristine Ag foil presented a flat surface (Supplementary Fig. 4a) in the SEM image, and the surface seems to be insensitive to the electrochemical reduction environment of ECR since no obvious change was observed after a 2-h reaction (Ag foil-2-h) at an overpotential of 0.7 V (Supplementary Fig. 4b). The Ag-D catalyst, on the other hand, contained abundant spherical nanoparticles and grain boundaries^27,46^, as labelled by blue arrows in Fig. 1g, h. The HRTEM image (Fig. 1i) further confirmed the presence of twinning defects, indicated by a blue arrow. The unique morphology of Ag-D seems to be considerably stable under the ECR environment. The SEM images of both Ag-D after a 2-h reaction (Ag-D-2-h, Supplementary Fig. 4c) and after an 80-hour reaction (Ag-D-80h, Supplementary Fig. 4d) exhibited a similar morphology to that of the original Ag-D (Fig. 1g). A high density of (111) surface steps and terraces was frequently observed in the HRTEM images (Fig. 2e, f). Briefly, although Ag-D shares a similar crystal structure with Ag foil, their micromorphologies differ markedly, which suggests distinct performances in ECR.

### Fast operando XAS analysis

Briefly, XANES provides reliable clues on an element’s oxidation state^33,47^, while EXAFS conveys local structural information surrounding the absorbing atom^28,35,48^. Although finding out a novel catalyst is an interesting task, it is much more important to understand the mechanism and the formation process of active sites in catalysts during ECR. Fast operando XAS was performed at the 44 A beam line in Taiwan Photon Source (TPS) to obtain information about the catalyst evolution during ECR. Equipped with a quick-scanning monochromator (Q-Mono), the hard X-ray (~4.5–34 keV) provided from the 44 A beam line can achieve a surprisingly superb time resolution and energy resolution. Specifically, 120 sheets of spectra at the absorption edge of interest for the elements of interest can be obtained per minute. By averaging these spectra, final spectra with excellent quality can be promised. Consequently, the 44 A beam line is qualified for time-dependent XAS studies, especially for the K-edge of 3*d* and 4*d* transition elements.

A home-made polymethyl methacrylate (PMMA) cell equipped with a three-electrode system allows the corresponding electrochemical experiments (Fig. 2a, and Supplementary Fig. 5). A round hole with a diameter of 1 centimetre, sealed by polyimide tape, allowed penetration of the hard X-ray for the working electrodes. The applied overpotential for the working electrode was 0.7 V during the operando measurements. The XANES spectra of pristine Ag_2_O (denoted as Dry, Fig. 2b, and Supplementary Fig. 6a) and Ag_2_O after 0.1 M KHCO_3_ electrolyte circulated into the cell (Wet, Supplementary Fig. 6a) were the same as the spectrum of the Ag_2_O reference^49–51^. Once the cathodic potential was on, the XANES profiles went through a distinct change from the first minute to the fifth minute (1–5 min, Supplementary Fig. 6a). From the beginning of the sixth minute, the XANES spectra of Ag-D presented increasingly similar profiles with that of the Ag foil reference, and eventually, we could barely distinguish them after a 30-min reaction time (Fig. 2b and Supplementary Fig. 6a). This conversion process from Ag_2_O to Ag was further confirmed to be irreversible from the XANES spectra after the removal of potential control at the working electrode (labelled by down, Supplementary Fig. 6a).

The time-dependent EXAFS spectra, on the other hand, provided key clues on the local structural information for Ag-D. The pristine Ag_2_O (labelled by Dry in Fig. 2c) exhibited a distinct peak for the first shell of Ag–O bond, while a suppressed peak for the second shell of Ag–Ag bond. The similar result was also reported in previous works^29,52,53^. It was found that the peak related to the second shell of Ag–Ag bond is strongly reduced at above 25 °C as compared with −228 °C. Our fast EXAFS experiments were all performed at room temperature, undoubtedly causing severe thermal vibration of the second shell. Moreover, the small particle size may also contribute to the weak peak intensity of Ag–Ag bond for the pristine Ag_2_O^28^. Once the cathodic potential was applied, the peak of the Ag–O bond (~2.08 Å) in R space decreased rapidly within the first four minutes and became indistinguishable at the sixth minute (Fig. 2c). The peak of the Ag–Ag bond (~2.86 Å), on the other hand, struggled to appear at the fourth minute, quickly grew from the sixth minute, and eventually became clear and distinguishable (Fig. 2c). These visible changes in peak position and intensity again qualitatively confirmed the conversion of Ag_2_O driven by the electrochemical reaction. Furthermore, the quantitative comparison of the time-dependent peak intensity for Ag–O and Ag–Ag bonds was visualized (Fig. 2d) by extracting EXAFS data in R space. The peak intensity of the Ag–Ag bond for Ag-D reached a stable value (~1.19) after 11 min. However, this value was less than the peak intensity of the Ag–Ag bond for the Ag foil reference (~1.33), indicating a possibly lower coordination number of Ag atoms in the Ag-D catalyst^32,54,55^.

This conclusion was validated by the fitting results for the time-dependent EXAFS spectra. The fitting parameters (Supplementary Table 1) and fitting plots in R space (Fig. 2c, and Supplementary Fig. 6b) and k-space (Supplementary Fig. 7) all guaranteed the reliable quality of the fitting outcomes. It is noted that the fitting path length was up to ~6 Å for Ag-D after 6, 8 and 10 min. The fitting results of the bond lengths and coordination number (CN) were reasonable and consistent with previous reports^52,54^. As shown in Fig. 2c, the peaks at about 5.00 Å in the spectra of 6, 8 and 10 min were assigned to the third shell of Ag–Ag bond. The CN of the first shell of Ag–Ag bond (~2.87 Å) gradually increased with the reaction time and finally reached a stable value. As shown in the Supplementary Table 1, the average CN of Ag-D after 30 min of ECR (~7.2) was still much smaller than that of the standard Ag foil reference (face-centred cubic, CN = 12.0), demonstrating the presence of massive Ag defects inside the Ag-D sample. The low value of CN for Ag-D can also be well explained from the viewpoint of Federico^56^ et al., who showed a generalized CN of 7.5 for surface Pt atoms at the (111) plane. In our case, Ag (111) lattice planes not only frequently appeared in TEM images (Fig. 1i and Fig. 2e, f) but also exhibited the highest peak intensity in the XRD pattern (Fig. 1a) for Ag-D. As shown in Fig. 2g, two unit cells in the *c*-axis were selected to depict the coordinated environments of Ag atoms (blue balls) located on the (111) plane (indicated by an orange plane). The generalized CN of the atom labelled by “0” is (6 × 9 + 3 × 12)/12 = 7.5, obtained by weighting each first-nearest neighbour atom (labelled by 1~9) by its CN. Besides the CN, the Debye–Waller (DW) factor *σ*^2^ is another significant outcome from the fitting of EXAFS data. Generally speaking, the DW factor consists of two components, static disorder and thermal vibrations^57,58^. In the present work, only the contribution from the static disorder can be discussed, since our EXAFS spectroscopy were collected at the same temperature (~25 °C). As shown in Supplementary Table 1, the *σ*^2^ for Ag foil and Ag-D (30 min) are 8.9 × 10^-3^ Å^2^ and 6.8 × 10^-3^ Å^2^, respectively, indicating the fluctuation of Ag–Ag bond lengths for the former is more violent than that for the latter^59^. This result is reasonable considering their different coordination numbers. For the Ag foil with the complete coordinated state (CN = 12.0), the local tiny fluctuation in Ag–Ag bond lengths could easily spread into the whole system, while for the Ag-D (30 min) with massive defects (CN = 7.2), the diffusion process of the fluctuation could be blocked around defect sites. In conclusion, the fitting results of the EXAFS spectra are reasonable and in accordance with the TEM and XRD results.

The defective structures in catalysts have been widely demonstrated to have a significant effect on their catalytic performance^3,60^. Several spectroscopy methods and microscopic imaging technologies have been reported as the useful tools to study the defects in catalysts^60^. For instance, Raman spectroscopy, which can detect the surface chemical bonds’ vibration signal, is able to quantitatively study the oxygen vacancies^61^ and carbon defects^62^ for carbon-based materials. Despite these achievements, the effective method to quantify the defects for metals and oxides remains to be developed. Recently, Wang et al. suggested that the change of CN for metal atoms from EXAFS data could be helpful in the analysis of the defect concentration^60^. The fitting of CN from EXAFS data has been successfully reported in our previous study^63^. Based on the knowledge, we introduce a concept, the relative concentration of defects (RC_d_), to perform a quantification analysis. The RC_d_ is defined as a ratio, where the numerator is the difference in coordination numbers between the studied sample and reference sample, and the denominator is the coordination number for the normal reference sample. Therefore, the RC_d_ for our Ag-D catalyst is (12.0 − 7.2)/12.0 = 40%, while for the Ag foil reference, this value can be ignored. In summary, we believe that these massive defects and the unique nanostructured morphology undertake a significant role in the ECR process^3,23,64^.

### Electrochemical performance of ECR

The performance evaluation of the catalysts was first carried out in an H-cell reactor using 0.1 M potassium bicarbonate electrolyte at room temperature (~25 °C). Argon (Ar) and CO_2_ was purged into the electrolyte, respectively, to compare the linear sweep voltammetry (LSV) results. As shown in Supplementary Fig. 8, the current density of the Ag-D catalyst in CO_2_-saturated solution is larger than that in Ar-saturated solution, roughly indicating its activity towards ECR.

**Fig. 3:**
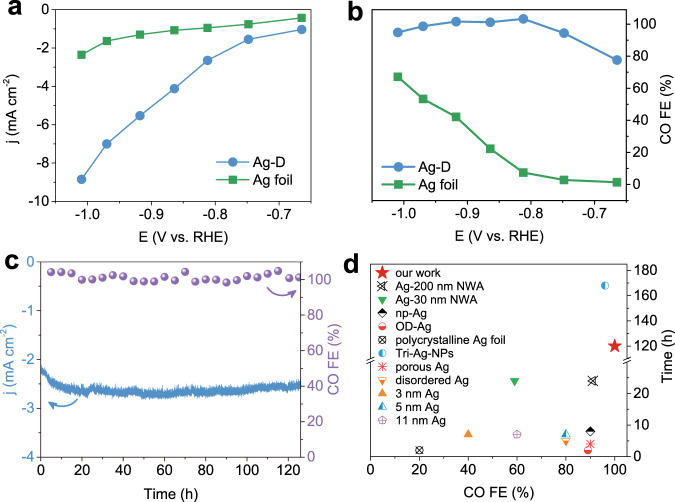
Electrochemical measurements in an H-cell reactor. **a** Total current density (j) curves of Ag-D and Ag foil catalysts. **b** FE of CO for Ag-D and Ag foil. **c** Stability of the total current density and FE of CO for the Ag-D catalyst. **d** ECR performance comparison on stability and FE of CO for some representative Ag-based catalysts.

A set of constant-potential electrolysis experiments were then conducted on Ag-D and Ag foil catalysts to compare their activities and selectivity. The current density of Ag-D was initially high and gradually decreased to a stable value after tens of seconds, corresponding to the conversion of Ag_2_O to Ag-D, as discussed before. Afterwards, the Ag-D electrode presented superb electrocatalytic performance for ECR, as revealed in Fig. 3. A larger total current density (j) and CO partial current density (j_CO_) were observed for the Ag-D electrode than the Ag foil (Fig. 3a), indicating the higher activity of the Ag-D electrode. To elucidate the reasons for this conclusion, the electrochemical surface area (ECSA) of Ag-D and Ag foil were estimated (Supplementary Fig. 9) for the convenience of intrinsic activity comparison. Almost identical ECSA-normalized j_CO_ values were observed at low overpotentials for the two electrodes (Supplementary Fig. 10), suggesting that the difference in the apparent activities of the nanostructured Ag-D and polycrystalline Ag foil is primarily caused by different amounts of active sites^65^. This conclusion can be rationalized by the larger RC_d_ value of Ag-D than the Ag foil, further proving the benefit of defects for ECR. The ECSA-normalized j_CO_ at high overpotentials, however, exhibited a lower value for Ag-D than for Ag foil, which was attributed to the limitation of mass transfer^65,66^.

As shown in Fig. 3b, the Ag-D catalyst displayed a nearly 100% FE for CO at −0.81 V, while a negligible amount of H_2_ product was detected on the gas chromatograph. Moreover, an extremely high FE was maintained even when the cathodic potential reached −0.96 V. In contrast, the electrode of Ag foil failed to reach 80% FE for CO from −0.66 to −1.01 V. It should be mentioned that no liquid products were detected in our study. The improved selectivity for CO of the Ag-D catalyst was ascribed to its nanostructured morphology and massive defects (RC_d_ = 40%), confirmed by SEM, TEM and operando XAS results, as mentioned before. Atoms surrounding defect positions are usually in a metastable state with unsaturated coordinated numbers, and thus possess a unique absorption capacity and catalytic property compared to stable atoms in the inner part of a crystalline grain^27,67–69^. The formation of defects in Ag-D was further investigated. Specifically, the original Ag–O bonds in Ag_2_O were broken by electron attack, and abundant Ag atoms were created and spontaneously combined with each other, forming nanocrystals with defect structures. We then assumed that the CO_2_ in the electrolyte could efficiently absorb onto atoms around the defects and thus prevented these active sites from combining or agglomerating with each other. To examine this supposition, we purged pure argon gas in place of CO_2_ into the electrolyte at the beginning of the electrochemical reduction reaction. After that, Ar gas was replaced by CO_2_ to test the ECR performance. The results of this controlled experiment are labelled as ‘Ar-CO_2_’ to distinguish them from the results of the normal experiment (labelled as ‘CO_2_’). The CO FEs of these two experiments had no obvious difference (Supplementary Fig. 11b), while their current density exhibited a difference (Supplementary Fig. 11a), indicating a possibly lower number of active sites for ‘Ar-CO_2_’ than for ‘CO_2_’. Therefore, we confirmed that CO_2_ in the electrolyte was helpful for the initial formation of massive defect structures in Ag-D to some extent.

The ECR performance of the Ag-D catalyst was also compared with the commercial Ag powder. The phase structure of Ag powder was confirmed from XRD data as seen in Supplementary Fig. 12a. The morphology of the Ag powder, as shown in Supplementary Fig. 12b, presented spherical particles in micron-level scale. The activity and selectivity of Ag powder were tested and the results were compared with Ag-D as exhibited in Supplementary Fig. 13. Obviously, both the total current density and FE of CO for Ag powder was lower than that for Ag-D, confirming the unique performance of our defect-rich Ag-D catalyst again.

We conducted a time-resolved ECR performance measurement in the first 15 min corresponding to the time scale of fast operando XAS, aiming to uncover the specific correlation between the electrochemical phenomenon and the structures observed from XAS. The total current density (j_total_, Supplementary Fig. 14a) slightly decreased from the beginning to the ~10 min, which may be mainly attributed to the reduction process of Ag_2_O to Ag as deduced from the fast XAS results. After that, a steady current density was achieved, corresponding to the electrochemical conversion of CO_2_ to CO. During the whole time-resolved electrolysis, the FE of H_2_ could be negligible. However, the FE of CO displayed an impressing relationship with the coordination number (CN) extracted from the fast XAS spectra (Supplementary Fig. 14b). Specifically, a low CN (~1.8, Supplementary Table 1) for Ag–Ag path was initially observed at ~4 min, resulting from a tiny amount of Ag (0) in the generated Ag-D (4 min) catalyst. Meanwhile, the generated Ag-D (4 min) catalyst with low CN was proved to show a poor CO selectivity of 60%. From the ~4 min to ~8 min, the FEs of CO for the Ag-D catalyst increased along with the improved CNs for Ag–Ag path. Finally, a nearly invariable CN (~7) was achieved at the ~10 min (Supplementary Table 1, Supplementary Fig. 15), accompanied by a ~100% selectivity for CO in the time-resolved electroreduction (Supplementary Fig. 14b). The correlation manifested the significance of a proper defect structures to the selectivity of ECR to CO.

A long-term stability test was carried out for Ag-D at −0.81 V. As seen in Fig. 3c, Ag-D maintained an almost unchanged CO FE (~100%) and current density during a test period of >120 h. The mechanism for the impressive stability was further investigated by ex situ XAS measurements. The Ag-D sample after a 40-h ECR test exhibited a similar XANES profile as the Ag foil reference (Supplementary Fig. 16a). The coordination number, which was fitted to ~9.1 based on the EXAFS results, was still less than the coordination number of the Ag foil (Supplementary Fig. 16b). Combining the XAS analysis with the SEM and TEM results mentioned before (Fig. 1, Supplementary Fig. 4), we believed that the outstanding stability was a consequence of the robust nanostructure and the stable existence of defects. Furthermore, some representative Ag-based catalysts were collected from the literature and were compared with the Ag-D catalyst in terms of CO FE and stability (Fig. 3d, and Supplementary Table 2)^18,20,22,23,39,70^. It is obvious that Ag-D is in a leading position in this comparison. The superb selectivity and stability of Ag-D may guarantee its promise for practical applications.

The *η* and the cathodic energy efficiency (EE) of our Ag-D was also compared with other catalysts. As listed in Supplementary Table 3, Au-based and single-atom type catalysts exhibited lower *η* to achieve a similar FE of CO. However, the cost and the preparation methods of these materials may become obstacles to the practical application. Also, it seems that the low overpotential is relied on the high concentration electrolyte. Therefore, the overpotential of our Ag-D is considerably competitive among the listed catalysts considering the same electrolyte conditions. Besides, the cathodic EE of Ag-D was analyzed, as exhibited in Supplementary Fig. 17. The curve of EE as the function of the overpotential presented a volcano shape, and the highest EE of ~65.7% arrived at an overpotential of ~0.70 V. The EE declined to a little smaller value of ~63.9% at a lower overpotential of ~0.64 V and a lower FE of ~94.39%. Furthermore, the EE of Ag-D was compared with some other catalysts (Supplementary Table 3). The EE as a function of overpotential (Supplementary Fig. 18a) and CO FE (Supplementary Fig. 18b) were plotted, respectively. The squares represented catalysts from the literatures, while orange squares with a larger size indicated our Ag-D catalyst. The EE of Ag-D was located in the intermediate level among all the selected catalysts, indicating that the high EE could be hardly produced with the high FE or low overpotential alone. It is also noteworthy that the EE of Ag-D was in the leading level among the listed Ag-based catalysts. In summary, a compromise between the overpotential and FE should be pursed to ensure a maximum of the EE.

**Fig. 4:**
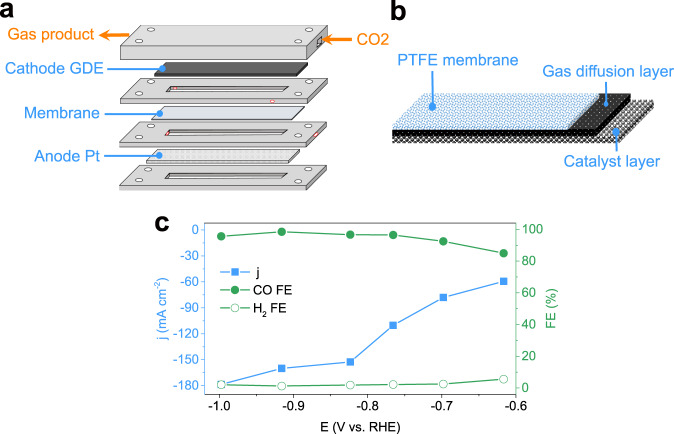
Electrochemical experiments in a flow-cell system. Schematic illustration of **a** microfluidic flow cell and **b** cathode GDE. **c** Total current density (blue) and FE of CO (green solid dots) and H_2_ (green hollow dots) as a result of controlling potentials.

The ECR performance of Ag-D was then tested in a flow-cell system to obtain a high reaction rate^44,65^. A high local pH has been demonstrated to be preferred for ECR over HER; thus, 1 M potassium hydroxide electrolyte was employed in the flow cell^1,65^. The structures of the flow cell and the cathodic GDE are exhibited in Fig. 4a and b, respectively. To fabricate the final cathodic GDE, an extra commercial polytetrafluoroethylene (PTFE) film with excellent hydrophobicity and breathability was mounted on top of the GDE. The electrocatalytic ability of the Ag-D GDE in the flow cell is shown in Fig. 4c. The FE for CO was 92–100% when the potential ranges from −0.7 to −1.0 V, agreeing with the results shown in the H-cell reactor. More importantly, the total current density of Ag-D achieved in the flow cell was up to ~180 mA cm^−2^ at a potential of −1.0 V. There was a distinct inflection point in the current density’s curve at about −0.82 V, which we believed was owing to limitations of mass and charge transfer at potentials more negative than this point. The structures of the inner flow channels and GDE and the operating parameters of flow cells (e.g. flow rate of CO_2_ and electrolyte) should be jointly optimized in future work to obtain better performance^44,71^.

### Theoretical study

**Fig. 5:**
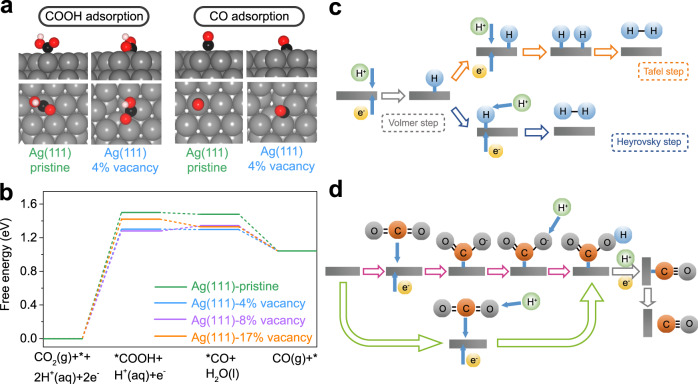
Theoretical study for assessing improved ECR performance. **a** The most preferred binding geometries of adsorbate on pristine Ag (111) and 4% vacancy-defected Ag (111). Side view: above; top view: below. Colour codes: Ag, grey; C, black; O, red; H, pink. **b** Calculated free energy diagrams. Proposed reaction pathways for **c** HER in acidic solutions and **d** ECR to CO on the surface of electrodes.

DFT calculations were performed to elucidate the reaction mechanism. A complete Ag (111) slab model is first constructed to simulate the intact Ag structure. Considering that different vacancy concentrations (C_v_, see the methods part for the definition) might lead to various structural fluctuations of the catalysts, which possibly affect the catalysis performance, we built three point defects carrying vacancy models with different C_v_ as representatives of defect structures (Supplementary Fig. 19) to investigate their influence on ECR. The smallest adsorption energy (∆*E*_ads_) of COOH is −2.0 eV on the pristine Ag (111) surface. The most stable binding geometry of COOH is at the top site, in which the C atom is on top of the Ag atom and the O–C–O plane is almost perpendicular to the metal surface (Fig. 5a). The local minima of CO at all four high symmetry sites can be located on the pristine Ag (111) surface. The top site and the hollow sites have the same ∆*E*_ads_ of −0.39 eV, which is 0.01 eV lower than the bridge site, agreeing with experimental observations that CO stably adsorbs at the top site of Ag^72^. Our ∆*E*_ads_ is comparable with the lowest ∆*E*_ads_ at the top site (−0.32 eV) reported by Chen et al. using the PW91 functional^73^. In the most preferred adsorption geometry, the C–O line is almost perpendicular to the metal surface (Fig. 5a), which is consistent with a previous study^73^. On the Ag (111)-4% vacancy surface (see the methods part for the definition), COOH stably binds at the vacancy edge site (Fig. 5a), in which the C atom and the carbonyl O atom interact with two Ag atoms separately (C–Ag distance: 2.15 Å, O–Ag distance: 2.47 Å) and the O–C–O plane tilts toward the vacancy central area. This geometry leads to a ∆*E*_ads_ of −2.20 eV, revealing stronger COOH binding than on pristine Ag surface. Similar to COOH, the ∆*E*_ads_ of CO is −0.57 eV on the Ag (111)-4% vacancy surface, demonstrating a stronger adsorption than that on the pristine Ag surface. CO prefers to bind at edge sites of the vacancy (Fig. 5a), where one C atom interacts equally with two Ag atoms (C–Ag distances: 2.23 Å) and CO tilts toward the vacancy central area. As shown in Supplementary Fig. 20, on Ag (111)-8% vacancy surface, the C atom and two O atoms of COOH interact with three Ag atoms separately (C–Ag distance: 2.17 Å, carbonyl(O)-Ag distance: 2.54 Å, hydroxyl(O)-Ag distance: 2.97 Å), and the O–C–O plane tilts toward the vacancy center area, resulting in a ∆*E*_ads_ of −2.24 eV. On Ag (111)-17% vacancy surface, the O–C–O plane of COOH is almost parallel to the Ag (111) surface over the vacancy, holding by the H–Ag (H–Ag distance: 2.70 Å) and C–Ag (C–Ag distance: 2.15 Å) interactions with the ∆*E*_ads_ of −2.09 eV. The binding geometry of CO on Ag (111)-8% vacancy surface is similar with that on pristine Ag (111) surface with a slightly lower ∆*E*_ads_ of −0.47 eV. On Ag (111)-17% vacancy surface, CO almost lies over the vacancy, showing a ∆*E*_ads_ of −0.56 eV.

To evaluate the influence of changes in COOH and CO adsorption properties on ECR to CO, the thermodynamic properties of the following reactions were computed.

$${\mathrm{CO}}_2\left( {\mathrm{g}} \right) + {\mathrm{H}}^ + + {\mathrm{e}}^ - + \ast \to \ast {\mathrm{COOH}}\quad \left( {{\mathrm{re}}.\;1,\;{\mathrm{activation}}\;{\mathrm{process}}} \right)$$

$$\ast {\mathrm{COOH}} + {\mathrm{H}}^ + + {\mathrm{e}}^ - \to \ast {\mathrm{CO}} + {\mathrm{H}}_2{\mathrm{O}}\left( {\mathrm{l}} \right)\quad \left( {{\mathrm{re}}.\;2,\;{\mathrm{surface}}\;{\mathrm{reaction}}} \right)$$

$$\ast {\mathrm{CO}} \to {\mathrm{CO}}\left( {\mathrm{g}} \right) + \ast \quad \left( {{\mathrm{re}}.\;3,\;{\mathrm{desorption}}\;{\mathrm{process}}} \right)$$

It is noted that the existence of intermediate COOH has been proved by the experimental evidence^74^. Moreover, the above reaction pathway has been accepted and discussed in abundant reports^20,25,54,75,76^. Therefore, it is reasonable to perform the DFT calculations on the basis of the above pathway in the present study. Figure 5b shows the calculated free energy diagrams at 0 V (Supplementary note 2) along the reaction pathway (pH = 6.8). The re. 1 is uphill on both the Ag (111)-pristine and vacancy surfaces with the larger reaction free energies within the two electrochemical reaction steps, revealing that the activation of carbon dioxide by protonation to form *COOH is the potential determining step. This is consistent with a previously reported conclusion on the Au (111) surface^76^. The strengthened binding of COOH results in higher limiting potentials (*U*_L_) on the vacancy containing surfaces (−1.30, −1.28 and −1.42 V for the 4%, 8% and 17% vacancy models, respectively) than that on the pristine surface (−1.5 V). Therefore, Ag vacancies can reduce the overpotential of ECR to CO prominently. The relatively weak binding of CO on Ag (111)-8% vacancy surface (−0.47 eV) leads to a slight energy rise (0.06 eV) for generating the *CO species, which, however, does not change the potential determining step of the whole reduction reaction. Among the three studied vacancy models, the Ag (111)-8% vacancy surface has the highest *U*_L_ (−1.28 eV), correspondingly the lowest overpotential. This indicates that there might exist an optimal concentration of defects, which can improve the catalytic performance of Ag to the largest extent. Further experimental work may be inspired to tune the defect concentrations of the catalysts for a better ECR performance.

In addition to the defect concentrations, we additionally built the Ag (100) and Ag (110) surface models to further confirm the benefits of defect structures on ECR performances (Supplementary note 3), considering the polycrystal property of the catalyst. The Ag (111), (100) and (110) crystal surfaces were corresponding to the three intensive peaks observed in the XRD pattern (Fig. 1a). As shown in Supplementary Figs. 22–24, the calculated results on the Ag (100)-pristine, Ag (100)-vacancy, Ag (110)-pristine and Ag (110) vacancy surfaces showed improved adsorption configuration of key intermediate COOH surrounding the defect sites. Therefore, the activation of CO_2_ on surfaces with Ag defects was thermodynamically more favorable than that on the pristine surfaces.

The HER is a main competing side reaction for ECR due to their overlapping potential windows^17^. Although the DFT calculations demonstrated the strengthened binding of intermediate COOH by defect sites, which accounts for the enhanced FE for ECR to CO, the mechanism for the dramatically inhibited HER is still ambiguous. Examining the possible reaction pathways of HER and ECR to CO may provide some useful clues. As shown in Fig. 5c, it is believed that HER in acidic electrolytes begins from a Volmer reaction, where an electron transfer is coupled with a proton adsorption on an empty active site of the electrode. After the formation of an absorbed hydrogen atom, two different reaction processes, the Tafel reaction or Heyrovsky reaction, may occur to yield the final H_2_ product^77^. In contrast, the reaction pathways for ECR are quite complex due to the multi-electron and multi-proton transfer steps. For ECR to CO, the COOH is generally regarded as a key intermediate^74,78^, which is also reported in our present work. According to Kortlever et al., the formation of *COOH may have two different mechanisms^78^, as shown in Fig. 5d. In the two-step mechanism (purple arrows), the electron and proton transfer processes occur in two separate steps, while in another mechanism (light green arrows), the proton transfer is coupled with the electron transfer (PCET). Regardless of the initial step, the formed *COOH will subsequently react with another electron and proton to yield H_2_O and *CO. Then *CO desorbs from the electrode to release CO and leaves an empty active site^39^. As shown in Fig. 5a, on the defect Ag surface, the COOH and CO stably bind at the vacancy edge sites where two Ag atoms are occupied, which likely blocks the adsorption of protons to Ag, leading to the poor selectivity of the HER.

## Discussion

In summary, the nanostructured Ag-D catalyst exhibits outstanding performance for ECR to CO, both in an H-cell reactor and a flow-cell reactor. Fast operando XAS measurements and ex situ SEM and TEM images revealed that massive defects are efficiently created at the initial stage of ECR. Carbon dioxide in the electrolyte is proved to undertake a role in the protection of these defect sites since their formation. We proposed the concept of the RC_d_ based on the coordination numbers to quantify the defects, which may give insights to the future work on the quantitative discussion of the defects, especially for the metal and metallic oxides. DFT calculations demonstrated that the adsorption of intermediate COOH is strengthened on Ag atoms surrounding the defect sites, benefiting the activation of CO_2_. Therefore, combining the experimental and theoretical results, the origin of the high performance of Ag-D catalyst was attributed to the abundant defect sites. Our work not only highlights the importance of operando characterization techniques with high time resolution, but also provides new insights into Ag-based catalysts for ECR.

## Methods

### Sample preparation

The Ag_2_O nanoparticles were synthesized by a precipitation reaction. In detail, 0.02 mol AgNO_3_ was first dissolved in a volume of 200 mL deionized water. Then, a volume of 100 mL sodium hydroxide solution (0.2 M) was dropped in the AgNO_3_ solution under stirring to enable a homogenous mixture. The final product of Ag_2_O was collected after filtration and drying at ~60 °C. Ag-D was prepared by operando electrochemical reduction of Ag_2_O, which endowed massive defects in Ag-D. Polycrystalline Ag foil (0.127-mm thick, 99.9%, annealed) was purchased from Alfa Aesar. Ag powder was purchased from Aladdin.

### Characterization

XRD was conducted on a Rigaku Smartlab with filtered Cu Kα radiation (λ = 1.5418 Å) to determine phase constitutions and crystal structures. XPS was carried out on a PHI5000 VersaProbe spectrometer using an Al-Kα X-ray source to analyse element states of the electrocatalysts. SEM images were obtained from a scanning electron micro-analyzer (HITACHIS4800). TEM images (JEOL JEM 2100, 200 kV) were collected to uncover catalysts’ morphology and structure.

### Electrode preparation

The electrode ink for Ag-D was prepared as follows. Pristine Ag_2_O powder (10 mg) and Super P Li (10 mg) were suspended in a mixture containing 5 wt% Nafion solution (100 μL) and ethanol (1 mL) via ultrasound. Then, 30 μL of the above homogeneous ink was spread on a prepolished L-type GCE with a diameter of 8 mm. The loading of Ag_2_O in this electrode was ~0.543 mg cm^−2^. Polycrystalline Ag foil served as the working electrode for comparison after thorough ultrasonic cleaning in acetone and ethanol.

For the fabrication of the cathodic GDE, the same ink was dropped onto the gas diffusion layers (29BC, SGL) with an Ag_2_O loading of ~2 mg cm^−2^. To avoid the possible ‘flooding’ of GDE, another commercial PTFE film (~0.22 μm pore size) was fitted on top of the GDE.

### ECR performance measurements and product analysis

Electrochemical tests were conducted on a workstation (CHI 760E) at 25 °C. A gas-tight H-cell reactor segregated with a Nafion-117 membrane was used for the electrolysis experiments. In the cathodic chamber (100 mL), an L-type GCE and Ag/AgCl served as the working and reference electrode, respectively. A Pt foil in the anodic chamber (100 mL) served as a counter electrode. Each chamber contained 50 mL KHCO_3_ (99.99%, Macklin) electrolyte (0.1 M, pH = 6.8). Carbon dioxide was continuously purged (30 mL min^−1^, 99.999%, Shangyuan) during the ECR process. All potentials were recalculated into RHE by *E*_*RHE*_ = *E*_*Ag/AgCl*_ + *0.1989* + *0.0591* × *pH* according to Nernst equation unless otherwise stated^54,65^, and iR compensation was also taken into consideration.

For the measurements in the flow cell, the as-prepared cathodic GDE (geometric active area of 1 cm^2^) was separated from the anode with a commercial anion exchange membrane (PK130, Fumatech). A Pt plate acted as the anode. Since 1 M KOH electrolyte was used, the Hg/HgO electrode was mounted in the catholyte stream at a fixed location, serving as the reference electrode. All measured potentials vs. Hg/HgO were recalculated into RHE through *E*_*RHE*_ = *E*_*Hg/HgO*_ + *0.098* + *0.0591* × *pH* according to the Nernst equation unless otherwise stated. The catholyte (~30 mL) was circulated by a peristaltic pump (BT100-2J, Longer).

Gaseous products were analyzed by a gas chromatograph (GC, HOPE 9860) with a thermal conductivity detector (TCD) and flame ionization detectors (FID). Nuclear magnetic resonance spectroscopy (^1^H NMR, Bruker ACF-400) was used to analyze the liquid-phase products.

The cathodic energy efficiency (EE) was calculated as follows^79,80^:

1 $${\mathrm{EE}}\left( \% \right) = \frac{{1.23 - {\mathrm{E}}^0}}{{1.23 - {\mathrm{E}}}} \times {\mathrm{FE,}}$$

where E^0^, FE and E represented standard potential, faradaic efficiency and applied potential, respectively.

### Fast operando XAS measurements

For the conventional XAS, it usually takes 15–30 min to obtain a single spectrum, and its quality may be unsatisfactory due to some accidental disturbances. Therefore, employing conventional XAS to study the chemical information of the catalyst may give confusing results, since the real state of the catalyst may change quickly with time. Moreover, the tracking of the changing process has been pursued and received growing attention in order to gain a comprehensive understanding on the reconstruction of catalysts^28^. Obviously, advanced characterization techniques with high time resolutions are essential for obtaining the desired information. The fast XAS technique introduced here can provide not only a clear evolution information on the catalyst, but also a reliable data quality, both of which are crucial for the further analysis on the structure of catalysts. Fast operando XAS was collected at the 44 A beam line of TPS. The 44 A beam line equipped with a quick-scanning monochromator (Q-Mono) can provide 120 sheets of spectra per minute. By averaging these spectra, a good quality of the final spectra can be ensured. A PMMA reactor was prepared for fast operando experiments, as shown in Supplementary Fig. 5. Catalysts were hand-brushed onto carbon-fibre paper serving as a working electrode. Carbon dioxide was constantly bubbled into the electrolyte (0.1 M KHCO_3_). A potential of −1.5 V vs. Ag/AgCl was applied to the working electrode to study the evolution of catalysts. All spectra were obtained in transmission mode and analyzed using Athena and Artemis^81^. The k range for Fourier transforms (FT) was from 2.5 to 12.2 Å^-1^. Fitting of the EXAFS spectra *χ*(k)k^2^ for Ag–O and Ag–Ag bond was conducted in R space in the range from R_min_ = 1.2 Å to R_max_ = 3.5 Å. When fitting the single peak for Ag–Ag bond, the R_min_ was set to 2.16 Å. For Ag-D after 6, 8 and 10 min, the high shells were fitted, respectively. The R_max_ was accordingly set to 6.0 Å. Phase corrections were taken into consideration during the fitting process.

### DFT calculations

To obtain a deeper insight into the effects of Ag defects on electrocatalytic reactions, DFT calculations were performed. In the current study, we built three vacancy models with point defects as representatives of defect structures to investigate their influence on ECR. The Ag (111) crystal surface is selected on the basis of the experimental observation and theoretical consideration. The Ag-D catalyst exhibited the strongest peak for (111) facet in XRD pattern, which was also frequently observed in HRTEM images. DFT calculations based on the selected Ag (111) plane was also reported in literatures for the mechanism studies^20,25,82^. Thus, it is reasonable to choose the representative Ag (111) facet to perform the DFT calculations in the present work. A three layer *p*(3 × 3) (111) slab with 20 Å of vacuum avoiding imaging interactions in the *z*-axis was constructed to represent the Ag pristine model. Meanwhile, the Ag (111)-vacancy system was built by removing one Ag atom from the optimized pristine Ag (111) system, and then further geometry optimization was performed to relax the system. Since the CN and DW factors reflect the coordination environment and structural disorder of the catalysts, accordingly, in the DFT studies, the influence of defects on the Ag (111) surface structures was also taken into consideration by optimizing lattice parameters and atomic positions of the slab. As shown in Supplementary Table 4, the existence of the vacancy does not change the cell shape of the slab, but leads to slight variation of the cell volume. This indicates that the vacancy gives rise to structural changes to some extent. For the clarity, the vacancy concentration is defined as ${\mathrm{C}}_{\mathrm{v}} = \frac{{{\mathrm{N}}_{\mathrm{v}}}}{{{\mathrm{N}}_{{\mathrm{total}}}}}$, where N_v_ is the number of vacancy, and N_total_ is the number of atoms of the first atomic layer in Ag (111)-pristine slabs. In this case, C_v_ = 1/27 ≈ 4%, and the model is referred to as Ag (111)-4% vacancy. The *p*(2 × 2) (111) slab models were constructed to simulate higher vacancy concentrations. By removing one and two Ag atoms, we obtained the models with C_v_ of ca. 8% and 17%, respectively (Supplementary Fig. 19). From Supplementary Table 4, we can see that when C_v_ is ca. 17%, the vacancies lead to slight variation of the cell shape compared with lower vacancy concentration carrying models, which indicates larger structural fluctuations with respect to the pristine structure. Note that, the C_v_ is not exactly equivalent to the RC_d_. The aim to introduce the C_v_ into DFT calculations is for the quantitative discussion on the effects of defect structures. For all models, the bottom layer was immobilized at the optimized lattice constant (2.03 Å), agreeing with the value of 2.04 Å obtained in experiments^83^. Initial adsorption configurations of COOH and CO intermediates at four sites (top site, bridge site, fcc hollow site and hcp hollow site, Supplementary note 1 and Supplementary Fig. 21) were constructed to study the adsorption properties.

The Vienna Ab-initio Simulation Package (VASP) was used to conduct periodic DFT calculations^84^. Electron-core interactions were described by the projector augmented wavefunction (PAW) method^85^. The Perdew-Burke-Ernzerhof (PBE) functional in a plane wave pseudopotential implementation within the generalized gradient approximation (GGA) was applied for describing the electron exchange-correlation^86^. Grimme’s semi-empirical correction for the dispersion potential (DFT-D3) was applied to include the long-range dispersion effect^87^. Plane wave cut-off energies of 700 eV and 450 eV were chosen for the bulk primitive crystal cell and slab models, respectively. The Monkhorst–Pack technique was used to automatically generate the gamma centred 3 × 3 × 1 *k*-points grid in the Brillouin zone^88^. The width of 0.1 eV was employed for Gaussian smearing. The electronic self-consistent iteration convergence criterion was 1.0 × 10^−6^ eV. The convergence criteria for the force was 0.01 eV/Å for each free atom.

## Supplementary information

Supplementary information

## Data Availability

All relevant data are available from the corresponding authors on reasonable request.

## References

[1] Dinh CT (2018). CO_2_ electroreduction to ethylene via hydroxide-mediated copper catalysis at an abrupt interface. Science.

[2] Singh MR, Goodpaster JD, Weber AZ, Head-Gordon M, Bell AT (2017). Mechanistic insights into electrochemical reduction of CO_2_ over Ag using density functional theory and transport models. Proc. Natl Acad. Sci. USA.

[3] Wang Y, Han P, Lv X, Zhang L, Zheng G (2018). Defect and interface engineering for aqueous electrocatalytic CO_2_ reduction. Joule.

[4] Clark EL (2018). Standards and protocols for data acquisition and reporting for studies of the electrochemical reduction of carbon dioxide. ACS Catal..

[5] AlOtaibi B, Fan S, Wang D, Ye J, Mi Z (2015). Wafer-level artificial photosynthesis for CO_2_ reduction into CH_4_ and CO using GaN nanowires. ACS Catal..

[6] Prajapati A, Singh MR (2019). Assessment of artificial photosynthetic systems for integrated carbon capture and conversion. ACS Sustain. Chem. Eng..

[7] Singh MR, Clark EL, Bell AT (2015). Thermodynamic and achievable efficiencies for solar-driven electrochemical reduction of carbon dioxide to transportation fuels. Proc. Natl Acad. Sci. USA.

[8] Hahn C (2017). Engineering Cu surfaces for the electrocatalytic conversion of CO_2_: controlling selectivity toward oxygenates and hydrocarbons. Proc. Natl Acad. Sci. USA.

[9] Singh MR, Kwon Y, Lum Y, Ager JW, Bell AT (2016). Hydrolysis of electrolyte cations enhances the electrochemical reduction of CO_2_ over Ag and Cu. J. Am. Chem. Soc..

[10] Singh MR, Clark EL, Bell AT (2015). Effects of electrolyte, catalyst, and membrane composition and operating conditions on the performance of solar-driven electrochemical reduction of carbon dioxide. Phys. Chem. Chem. Phys..

[11] Zhu DD, Liu JL, Qiao SZ (2016). Recent advances in inorganic heterogeneous electrocatalysts for reduction of carbon dioxide. Adv. Mater..

[12] Yang HB (2018). Atomically dispersed Ni(I) as the active site for electrochemical CO_2_ reduction. Nat. Energy.

[13] Ringe S (2020). Double layer charging driven carbon dioxide adsorption limits the rate of electrochemical carbon dioxide reduction on gold. Nat. Commun..

[14] Nitopi S (2019). Progress and perspectives of electrochemical CO_2_ reduction on copper in aqueous electrolyte. Chem. Rev..

[15] Gabardo CM (2018). Combined high alkalinity and pressurization enable efficient CO_2_ electroreduction to CO. Energy Environ. Sci..

[16] Gao D, Arán-Ais RM, Jeon HS, Roldan Cuenya B (2019). Rational catalyst and electrolyte design for CO_2_ electroreduction towards multicarbon products. Nat. Catal..

[17] Sun D, Xu X, Qin Y, Jiang SP, Shao Z (2020). Rational design of Ag-based catalysts for the electrochemical CO_2_ reduction to CO: a review. ChemSusChem.

[18] Deng W (2018). Achieving convenient CO_2_ electroreduction and photovoltage in tandem using potential-insensitive disordered Ag nanoparticles. Chem. Sci..

[19] Kuhl KP (2014). Electrocatalytic conversion of carbon dioxide to methane and methanol on transition metal surfaces. J. Am. Chem. Soc..

[20] Liu S (2017). Shape-dependent electrocatalytic reduction of CO_2_ to CO on triangular silver nanoplates. J. Am. Chem. Soc..

[21] Li YC (2019). Binding site diversity promotes CO_2_ electroreduction to ethanol. J. Am. Chem. Soc..

[22] Luan C (2018). High-performance carbon dioxide electrocatalytic reduction by easily fabricated large-scale silver nanowire arrays. ACS Appl. Mater. Interfaces.

[23] Lu Q (2014). A selective and efficient electrocatalyst for carbon dioxide reduction. Nat. Commun..

[24] Jia Y (2019). Identification of active sites for acidic oxygen reduction on carbon catalysts with and without nitrogen doping. Nat. Catal..

[25] Mistry H (2017). Enhanced carbon dioxide electroreduction to carbon monoxide over defect-rich plasma-activated silver catalysts. Angew. Chem. Int. Ed..

[26] Chen Y, Li WC, Kanan WM (2012). Aqueous CO_2_ reduction at very low overpotential on oxide-derived Au nanoparticles. J. Am. Chem. Soc..

[27] Li CW, Ciston J, Kanan MW (2014). Electroreduction of carbon monoxide to liquid fuel on oxide-derived nanocrystalline copper. Nature.

[28] Handoko AD, Wei F, Jenndy, Yeo BS, Seh ZW (2018). Understanding heterogeneous electrocatalytic carbon dioxide reduction through operando techniques. Nat. Catal..

[29] Firet NJ (2019). Operando EXAFS study reveals presence of oxygen in oxide-derived silver catalysts for electrochemical CO_2_ reduction. J. Mater. Chem. A.

[30] Zhu Y, Wang J, Chu H, Chu Y-C, Chen HM (2020). In situ/operando studies for designing next-generation electrocatalysts. ACS Energy Lett..

[31] Xiao, Z. et al. Operando identification of the dynamic behavior of oxygen vacancy-rich Co_3_O_4_ for oxygen evolution reaction. *J. Am. Chem. Soc*. **142**, 12087–12095 (2020). 10.1021/jacs.0c00257 32538073

[32] Lee JH (2019). Tuning the activity and selectivity of electroreduction of CO_2_ to synthesis gas using bimetallic catalysts. Nat. Commun..

[33] Sun H (2019). Ternary phase diagram-facilitated rapid screening of double perovskites as electrocatalysts for the oxygen evolution reaction. Chem. Mater..

[34] Bergmann A, Roldan Cuenya B (2019). Operando insights into nanoparticle transformations during catalysis. ACS Catal..

[35] Chen G (2019). An amorphous nickel–iron-based electrocatalyst with unusual local structures for ultrafast oxygen evolution reaction. Adv. Mater..

[36] Zhou J (2020). Voltage- and time-dependent valence state transition in cobalt oxide catalysts during the oxygen evolution reaction. Nat. Commun..

[37] Sun H (2020). Bulk and surface properties regulation of single/double perovskites to realize enhanced oxygen evolution reactivity. ChemSusChem.

[38] Tsai F-T (2020). The HER/OER mechanistic study of an FeCoNi-based electrocatalyst for alkaline water splitting. J. Mater. Chem. A.

[39] Ma M, Trześniewski BJ, Xie J, Smith WA (2016). Selective and efficient reduction of carbon dioxide to carbon monoxide on oxide-derived nanostructured silver electrocatalysts. Angew. Chem. Int. Ed..

[40] Fabbri E (2017). Dynamic surface self-reconstruction is the key of highly active perovskite nano-electrocatalysts for water splitting. Nat. Mater..

[41] Lin S-C (2020). Operando time-resolved X-ray absorption spectroscopy reveals the chemical nature enabling highly selective CO_2_ reduction. Nat. Commun..

[42] Peterson AA, Nørskov JK (2012). Activity descriptors for CO_2_ electroreduction to methane on transition-metal catalysts. J. Phys. Chem. Lett..

[43] Zhong M (2020). Accelerated discovery of CO_2_ electrocatalysts using active machine learning. Nature.

[44] Weekes DM, Salvatore DA, Reyes A, Huang A, Berlinguette CP (2018). Electrolytic CO_2_ reduction in a flow cell. Acc. Chem. Res..

[45] Tjeng LH (1990). Electronic structure of Ag_2_O. Phys. Rev. B.

[46] Feng X, Jiang K, Fan S, Kanan MW (2016). A direct grain-boundary-activity correlation for CO electroreduction on Cu nanoparticles. ACS Cent. Sci..

[47] Sun H (2019). Boosting the oxygen evolution reaction activity of a perovskite through introducing multi-element synergy and building an ordered structure. J. Mater. Chem. A.

[48] Liu H (2017). Insight into the role of metal–oxygen bond and O 2p hole in high-voltage cathode LiNi_x_Mn_2–x_O_4_. J. Phys. Chem. C..

[49] Chen P-T (2017). Size-selective reactivity of subnanometer Ag_4_ and Ag_16_ clusters on a TiO_2_ surface. J. Phys. Chem. C..

[50] Masai H (2019). X-ray absorption near-edge structure of Ag cations in phosphate glasses for radiophotoluminescence applications. J. Ceram. Soc. JAPAN.

[51] Buckley, J. J., Gai, P. L., Lee, A. F., Olivi, L. & Wilson, K. Silver carbonate nanoparticles stabilised over alumina nanoneedles exhibiting potent antibacterial properties. *Chem. Commun*. 4013–4015 (2008). https://pubs.rsc.org/--/content/articlelanding/2008/cc/b809086f/unauth#!divAbstract. 10.1039/b809086f 18758610

[52] Sarode PR (2002). Study of local environment of Ag in Ag/CeO_2_ catalyst by EXAFS. Mater. Res. Bull..

[53] Sanson A (2006). Negative thermal expansion and local dynamics in Cu_2_O and Ag_2_O. Phys. Rev. B.

[54] Li H (2019). Colloidal silver diphosphide (AgP_2_) nanocrystals as low overpotential catalysts for CO_2_ reduction to tunable syngas. Nat. Commun..

[55] Zhang, W. et al. Atypical oxygen-bearing copper boosts ethylene selectivity toward electrocatalytic CO_2_ reduction. *J. Am. Chem. Soc*. **142**, 11417–11427 (2020). 10.1021/jacs.0c01562 32506908

[56] Calle-Vallejo F (2017). Why conclusions from platinum model surfaces do not necessarily lead to enhanced nanoparticle catalysts for the oxygen reduction reaction. Chem. Sci..

[57] Ikemoto H, Miyanaga T (2007). Extended X-ray absorption fine structure study of local structure and atomic correlations of tellurium nanoparticles. Phys. Rev. Lett..

[58] Vila FD, Rehr JJ, Rossner HH, Krappe HJ (2007). Theoretical x-ray absorption Debye-Waller factors. Phys. Rev. B.

[59] Kenji K (2016). XAFS analysis of crystal GeCu_2_Te_3_ phase change material. Z. Phys. Chem..

[60] Xie C (2020). Defect chemistry in heterogeneous catalysis: recognition, understanding, and utilization. ACS Catal..

[61] Lee Y (2011). Raman analysis of mode softening in nanoparticle CeO_2−δ_ and Au-CeO_2−δ_ during CO oxidation. J. Am. Chem. Soc..

[62] Banhart F, Kotakoski J, Krasheninnikov AV (2011). Structural defects in graphene. ACS Nano.

[63] Guan D (2020). Utilizing ion leaching effects for achieving high oxygen-evolving performance on hybrid nanocomposite with self-optimized behaviors. Nat. Commun..

[64] Yan D (2017). Defect chemistry of nonprecious-metal electrocatalysts for oxygen reactions. Adv. Mater..

[65] Luo W, Zhang J, Li M, Züttel A (2019). Boosting CO production in electrocatalytic CO_2_ reduction on highly porous Zn catalysts. ACS Catal..

[66] Dunwell M, Luc W, Yan Y, Jiao F, Xu B (2018). Understanding surface-mediated electrochemical reactions: CO_2_ reduction and beyond. ACS Catal..

[67] Gavrilov AN (2007). On the influence of the metal loading on the structure of carbon-supported PtRu catalysts and their electrocatalytic activities in CO and methanol electrooxidation. Phys. Chem. Chem. Phys..

[68] Wang S, Jiang SP, White TJ, Guo J, Wang X (2009). Electrocatalytic activity and interconnectivity of Pt nanoparticles on multiwalled carbon nanotubes for fuel cells. J. Phys. Chem. C..

[69] Wang Y (2017). Layered double hydroxide nanosheets with multiple vacancies obtained by dry exfoliation as highly efficient oxygen evolution electrocatalysts. Angew. Chem. Int. Ed..

[70] Wang H (2016). Enhanced CO selectivity and stability for electrocatalytic reduction of CO_2_ on electrodeposited nanostructured porous Ag electrode. J. CO2 Util..

[71] Liu K, Smith WA, Burdyny T (2019). Introductory guide to assembling and operating gas diffusion electrodes for electrochemical CO_2_ reduction. ACS Energy Lett..

[72] McElhiney G, Papp H, Pritchard J (1976). The adsorption of Xe and CO on Ag(111). Surf. Sci..

[73] Chen BWJ, Kirvassilis D, Bai Y, Mavrikakis M (2019). Atomic and molecular adsorption on Ag(111). J. Phys. Chem. C..

[74] Firet NJ, Smith WA (2017). Probing the reaction mechanism of CO_2_ electroreduction over Ag films via operando infrared spectroscopy. ACS Catal..

[75] Ju W (2017). Understanding activity and selectivity of metal-nitrogen-doped carbon catalysts for electrochemical reduction of CO_2_. Nat. Commun..

[76] Gao D (2017). Enhancing CO_2_ electroreduction with the metal–oxide interface. J. Am. Chem. Soc..

[77] Guan D (2019). Screening highly active perovskites for hydrogen-evolving reaction via unifying ionic electronegativity descriptor. Nat. Commun..

[78] Kortlever R, Shen J, Schouten KJP, Calle-Vallejo F, Koper MTM (2015). Catalysts and reaction pathways for the electrochemical reduction of carbon dioxide. J. Phys. Chem. Lett..

[79] Liu S, Sun C, Xiao J, Luo J-L (2020). Unraveling structure sensitivity in CO_2_ electroreduction to near-unity CO on silver nanocubes. ACS Catal..

[80] Wang Y (2020). Catalyst synthesis under CO_2_ electroreduction favours faceting and promotes renewable fuels electrosynthesis. Nat. Catal..

[81] Ravel B, Newville M (2005). ATHENA, ARTEMIS, HEPHAESTUS: data analysis for X-ray absorption spectroscopy using IFEFFIT. J. Synchrotron Radiat..

[82] Feng L (2018). Structural identification of silicene on the Ag(111) surface by atomic force microscopy. Phys. Rev. B.

[83] Suh I-K, Ohta H, Waseda Y (1988). High-temperature thermal expansion of six metallic elements measured by dilatation method and X-ray diffraction. J. Mater. Sci..

[84] Kresse G, Furthmüller J (1996). Efficient iterative schemes for ab initio total-energy calculations using a plane-wave basis set. Phys. Rev. B.

[85] Kresse G, Joubert D (1999). From ultrasoft pseudopotentials to the projector augmented-wave method. Phys. Rev. B.

[86] Perdew JP, Burke K, Ernzerhof M (1996). Generalized gradient approximation made simple. Phys. Rev. Lett..

[87] Grimme S, Ehrlich S, Goerigk L (2011). Effect of the damping function in dispersion corrected density functional theory. J. Comput. Chem..

[88] Monkhorst HJ, Pack JD (1976). Special points for Brillouin-zone integrations. Phys. Rev. B.

