# Supplementary material for: Fast operando spectroscopy tracking in situ generation of rich defects in silver nanocrystals for highly selective electrochemical CO2 reduction
Source: Nat Commun. 2021 Jan 28;12:660. doi: 10.1038/s41467-021-20960-8 (PMC7844229; doi:10.1038/s41467-021-20960-8)
Supplement: Supplementary file 1 — Supplementary information [file 41467_2021_20960_MOESM1_ESM.pdf]

Supplementary materials

Fast operando spectroscopy tracking in-situ generation of rich defects in silver nanocrystals for highly selective electrochemical CO_2 reduction

Xinhao Wu#, Yanan Guo#, Zengsen Sun, Fenghua Xie, Daqin Guan, Jie Dai, Fengjiao Yu, Zhiwei Hu, Yu-Cheng Huang, Chih-Wen Pao, Jeng-Lung Chen, Wei Zhou * and Zongping Shao*

*Correspondence and requests for materials should be addressed to W.Z. (email: zhouwei1982@njtech.edu.cn) or to Z.S. (email: shaozp@njtech.edu.cn).

#These authors contributed equally to this work.

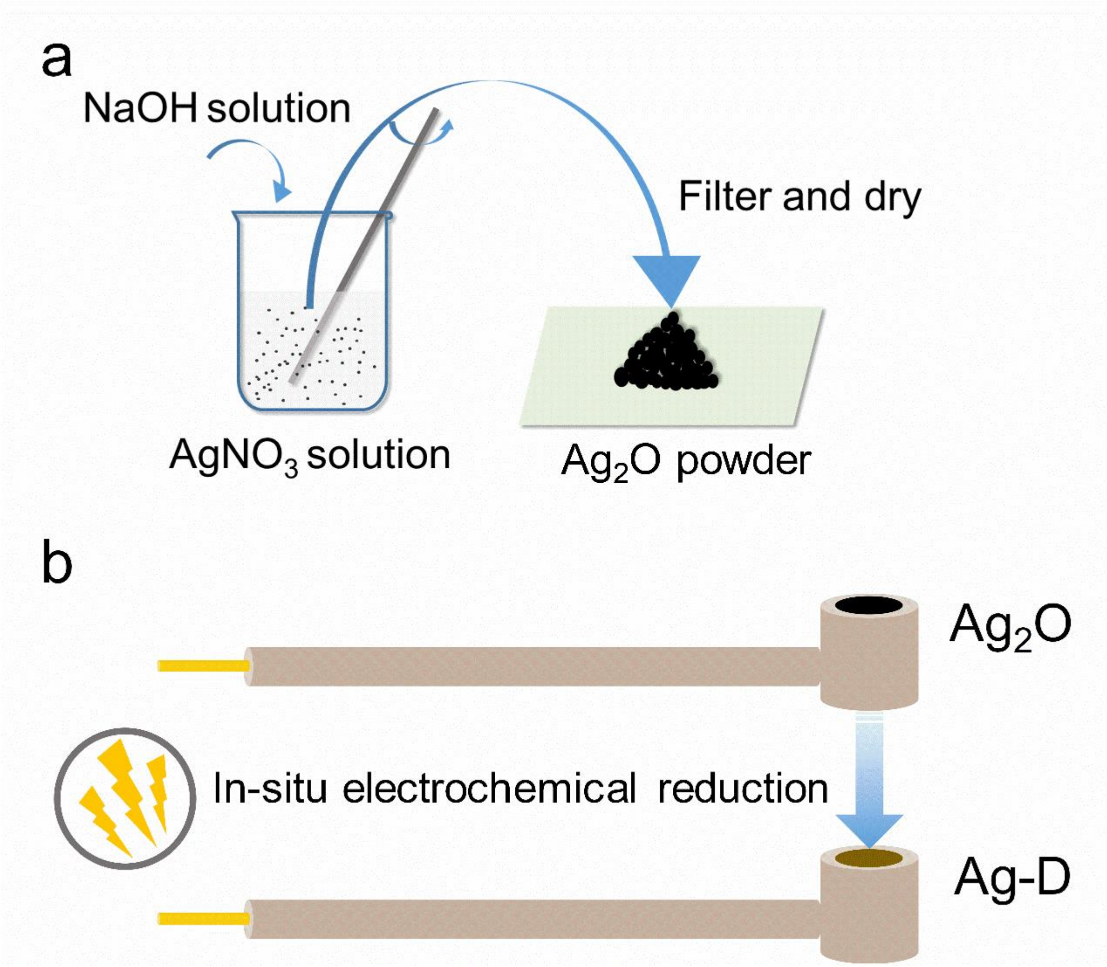

Supplementary Figure 1. Schematics of the preparation for Ag_2O and Ag-D catalysts. **a** The preparation of Ag_2O by a precipitation reaction. **b** Operando preparation of Ag-D catalyst by the electrochemical reduction process.

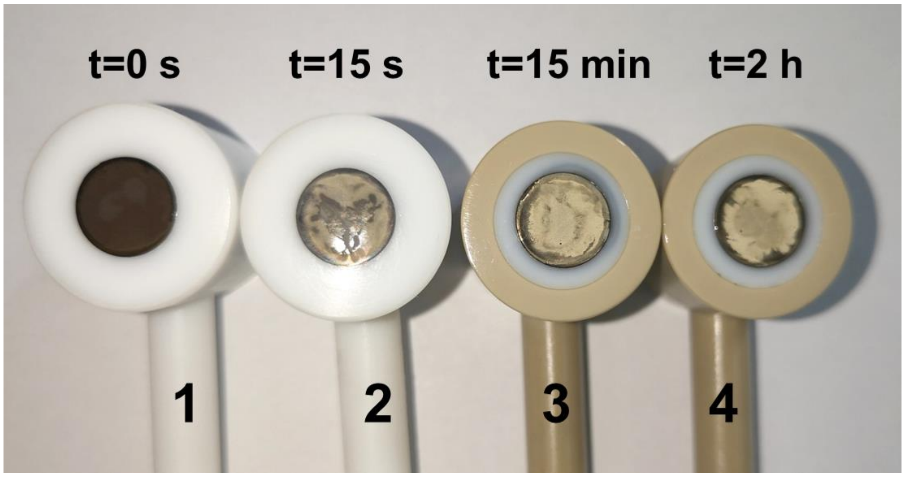

Supplementary Figure 2. Digital image showing the color changes of Ag-D electrodes along with reduction time. When t=0 s, the electrode's color was dark and it represented pristine pure Ag_2O. It is noteworthy that no carbon black was added in the ink under this case for the aim of a better observation and comparison.

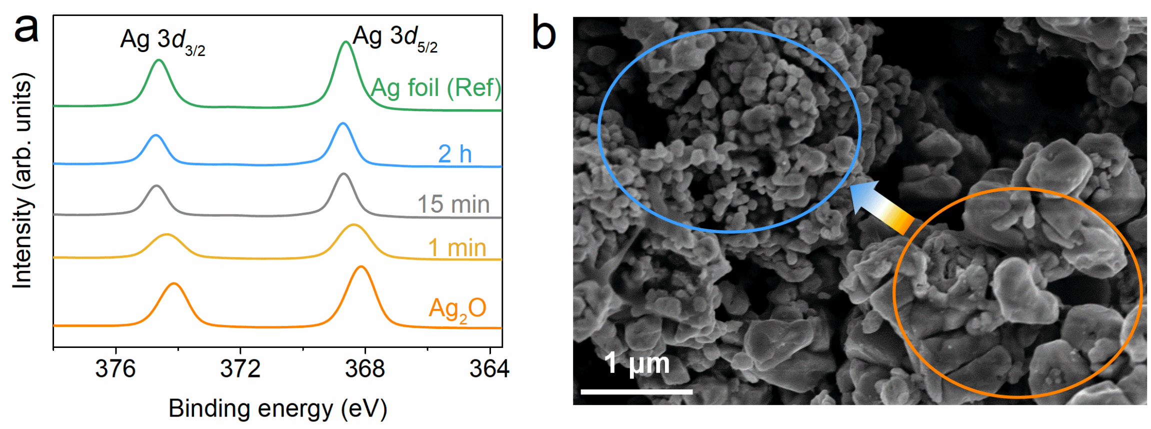

Supplementary Figure 3. Ex-situ characterization of the transformation process. **a** Time-dependent ex-situ XPS results of the catalyst, and Ag foil for reference. **b** SEM image of the catalyst after a 15-second reduction time. No carbon black was added in the ink under this case for a better observation.

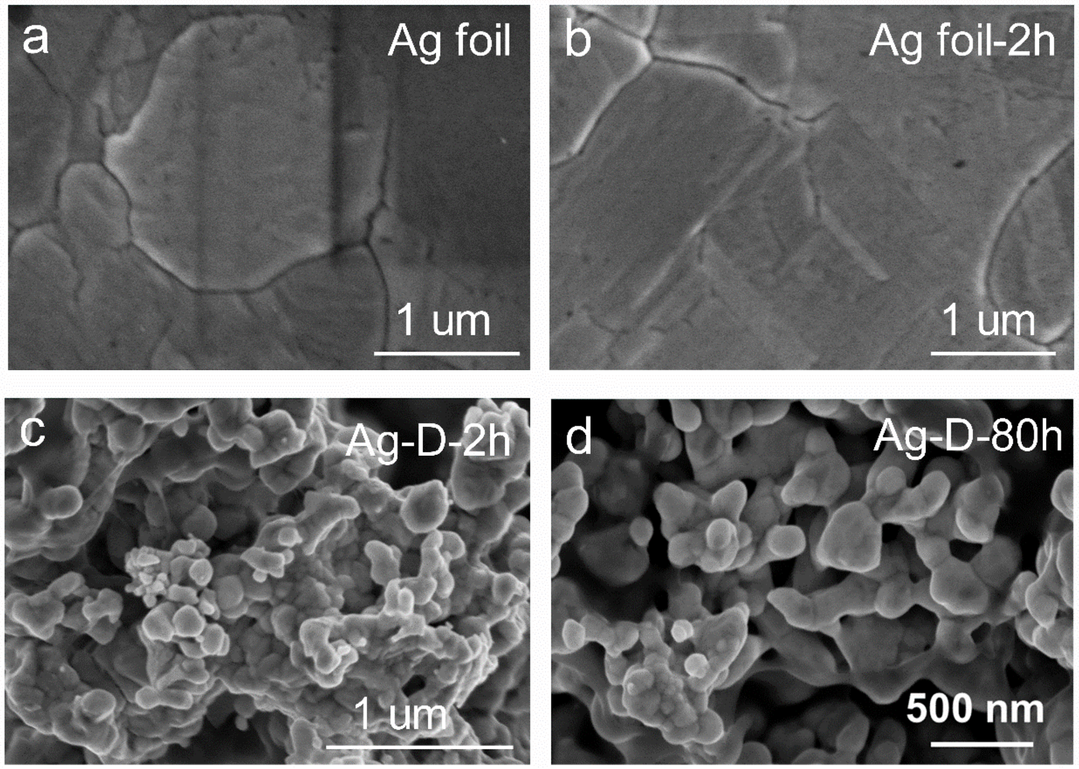

Supplementary Figure 4. Morphology analysis of Ag foil and Ag-D. SEM images of **a** pristine Ag foil, **b** Ag foil after 2-hour ECR reaction, **c** Ag-D after 2-hour reaction, and **d** Ag-D after 80-hour reaction. All these reactions were under the same overpotential of 0.7 V.

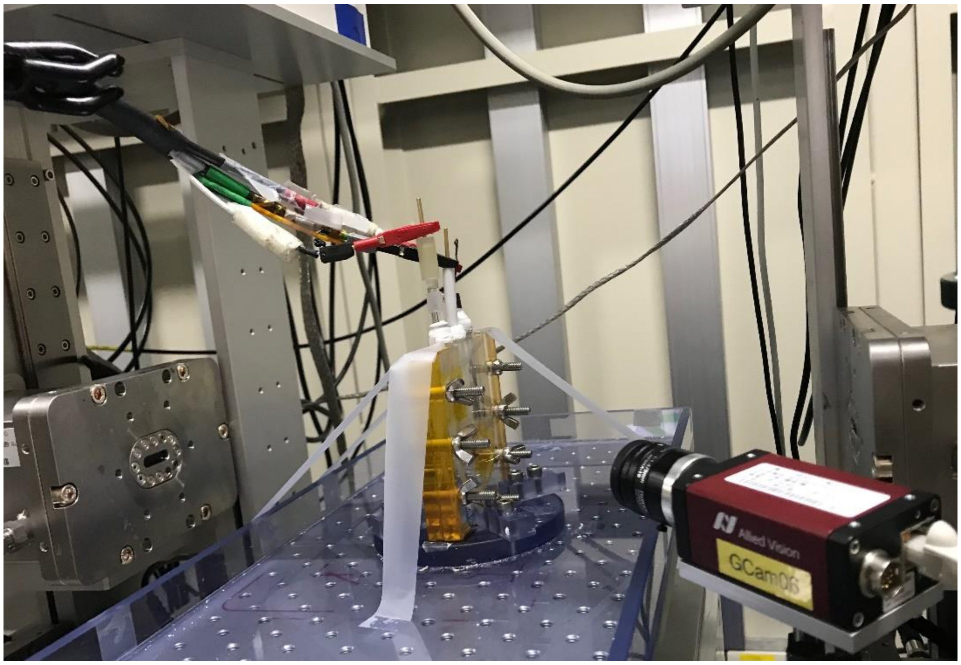

Supplementary Figure 5. Digital photograph of a home-made electrochemical cell for the operando XAS experiments.

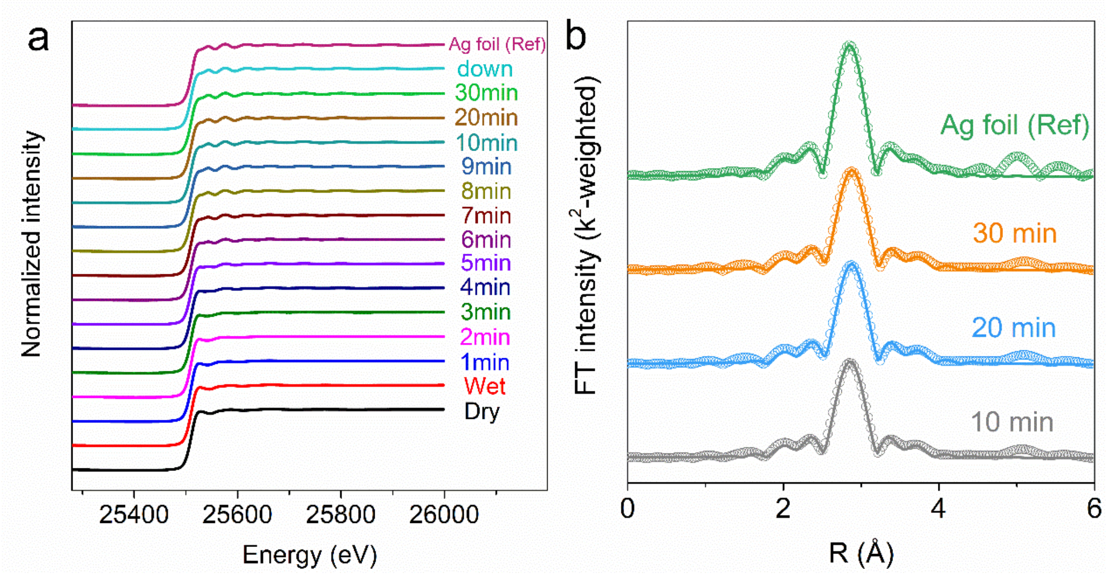

Supplementary Figure 6. Operando time-dependent XAFS results analysis. **a** Normalized XANES profiles for Ag-D and Ag foil reference. **b** k^2-weighted EXAFS spectra (hollow circles) and the corresponding fitting results (solid lines) for Ag-D with several different reaction times and Ag foil reference.

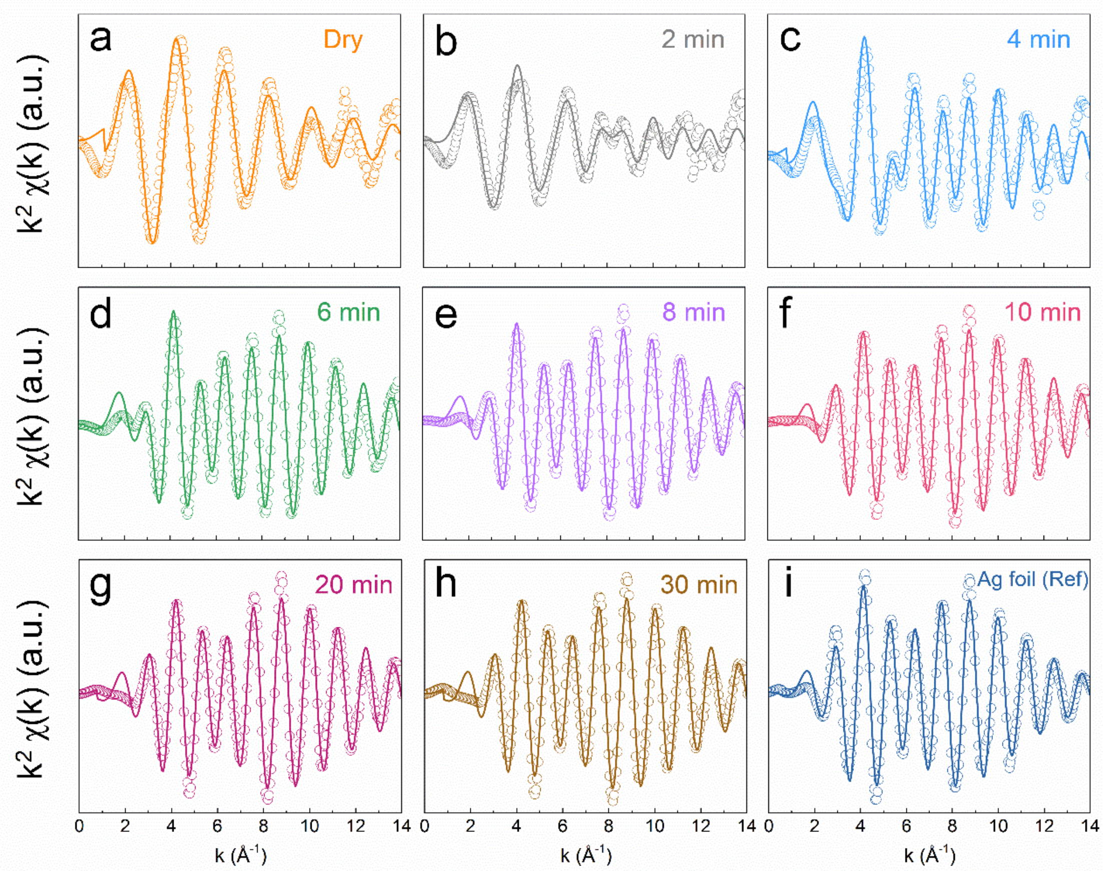

Supplementary Figure 7. The k space fitting results at Ag K-edge. **a** pristine Ag_2O. Ag-D after reaction times of **b** 2 min, **c** 4 min, **d** 6 min, **e** 8 min, **f** 10 min, **g** 20 min, and **h** 30 min. **i** Ag foil reference.

Supplementary Table 1. Fitting results of Ag K-edge EXAFS data for the Ag foil reference and time-dependent Ag-D catalyst.

Sample	Path	CN	R (Å)	σ^2 (10^{-3} Å^2)	ΔE_0 (eV)	R factor
Ag foil (Ref)	Ag-Ag	12.0	2.87 ± 0.002	8.9 ± 0.3	-0.5 ± 0.3	0.002
Dry	Ag-O	1.6 ± 0.25	2.06 ± 0.013	2.5 ± 1.6	4.8 ± 1.7	0.039
2 min	Ag-O	1.5 ± 0.30	2.09 ± 0.020	9.2 ± 3.0	0.4 ± 2.2	0.045
	Ag-Ag	0.2 ± 0.19	2.86 ± 0.018	2.8 ± 4.2		
4 min	Ag-O	1.2 ± 0.23	2.08 ± 0.012	7.2 ±2.7	2.5 ± 0.9	0.018
	Ag-Ag	1.8 ± 0.25	2.87 ± 0.006	6.3 ± 0.9		
6 min	Ag-Ag	4.9 ± 0.34	2.87 ± 0.003	7.1 ± 0.5	0.2 ± 0.4	0.009
	Ag-Ag	2.8 ± 1.53	4.02 ± 0.022	13.1 ± 5.0		
	Ag-Ag	2.1 ± 1.74	5.00 ± 0.016	5.3 ± 4.7		
8 min	Ag-Ag	6.7 ± 0.42	2.87 ± 0.003	7.2 ± 0.4	-1.8 ± 0.4	0.012
	Ag-Ag	3.4 ± 2.02	4.03 ± 0.022	12.7 ± 5.2		
	Ag-Ag	2.5 ± 2.29	5.00 ± 0.018	5.1 ± 5.0		
10 min	Ag-Ag	7.5 ± 0.46	2.87 ± 0.003	7.0 ± 0.5	-0.02 ± 0.42	0.007
	Ag-Ag	3.4 ± 2.13	4.03 ± 0.023	12.0 ± 5.3		
	Ag-Ag	2.6 ± 2.43	5.00 ± 0.017	4.6 ± 5.1		
20 min	Ag-Ag	7.2 ± 0.57	2.87 ± 0.004	6.8 ± 0.5	2.3 ± 0.5	0.006
30 min	Ag-Ag	7.2 ± 0.58	2.87 ± 0.004	6.8 ± 0.5	2.7 ± 0.5	0.006

CN: coordination number; R: distance between absorber and backscatter atoms; σ^2: Debye-Waller factor (DW) to account for both thermal and structural disorders; ΔE_0: inner potential correction; R factor: the goodness of the fit. The S_0^2 values, an amplitude reduction factor, was determined as 0.714 from the Ag foil reference.

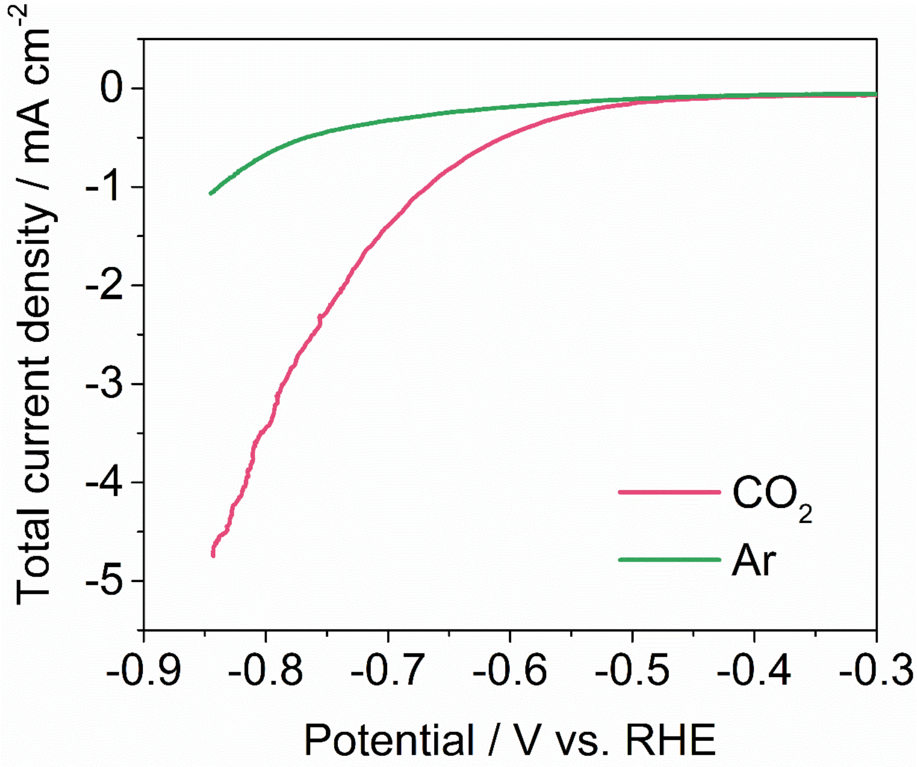

Supplementary Figure 8. LSV scans of Ag-D catalyst under CO_2- and Ar-purged 0.1 M $KHCO_3$ electrolyte, respectively.

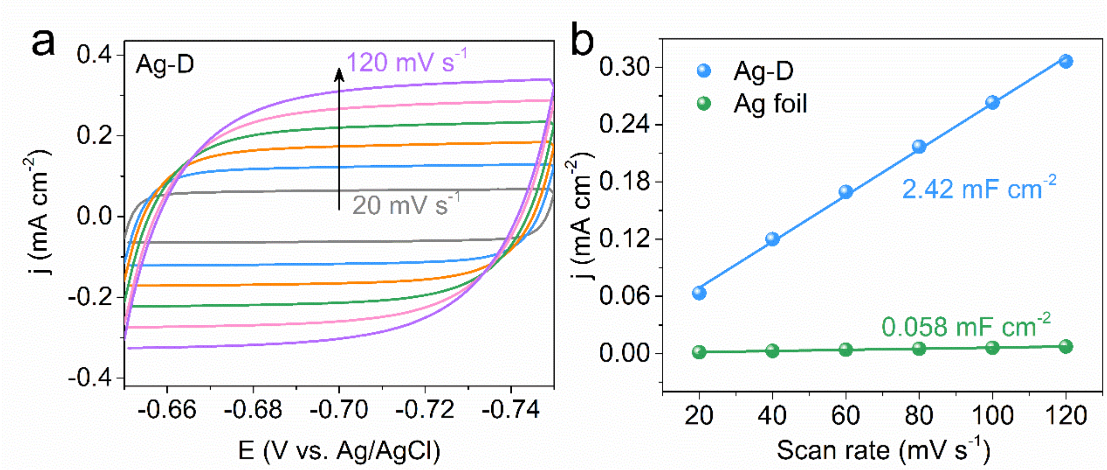

Supplementary Figure 9. ECSA estimation determined from C_{dl}. **a** CVs of Ag-D at a range of -0.65 V vs. Ag/AgCl (no iR-corrected) and -0.75 V vs. Ag/AgCl without faraday reaction. The scan rates are set as 20, 40, 60, 80, 100, and 120 mV s^{-1}. **b** Linear fitting of the capacitive currents versus scan rates for Ag-D and Ag foil electrocatalysts.

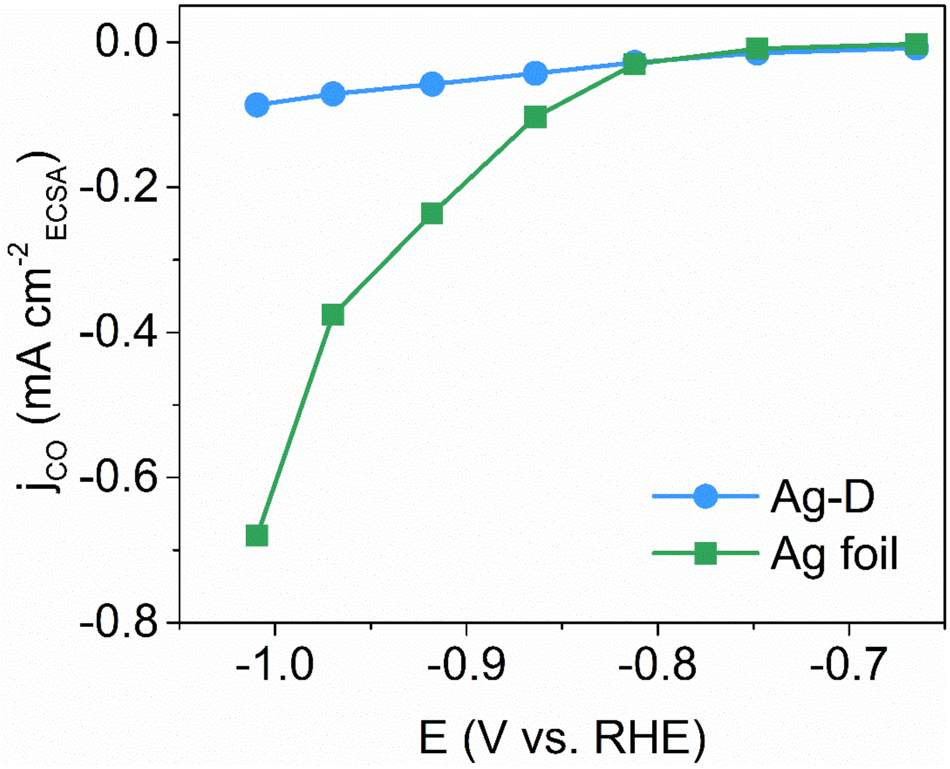

Supplementary Figure 10. ECSA-normalized CO partial current density for Ag-D and Ag foil electrodes.

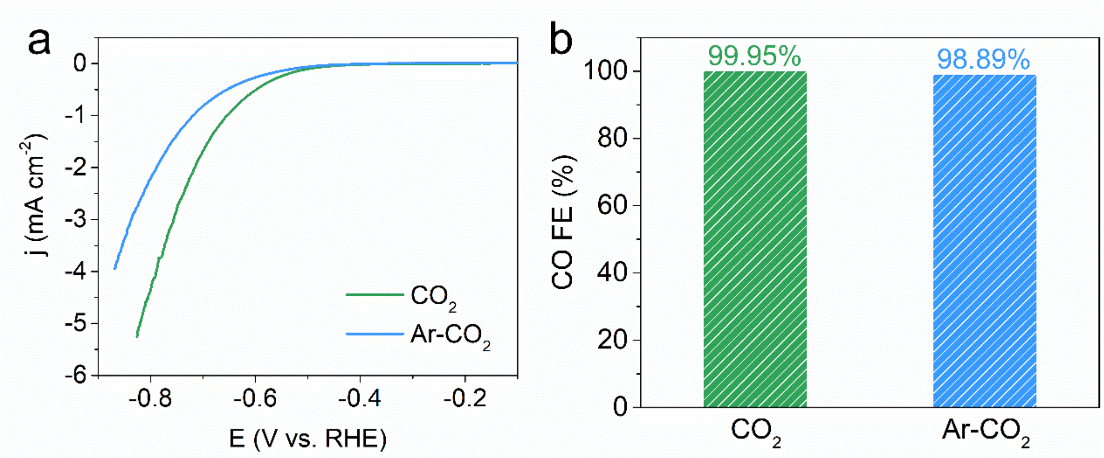

Supplementary Figure 11. Electrochemical test of the impact of CO_2 on the formation of defect. **a** Total current density of the normal experiment (CO_2) and the controlled experiment (Ar-CO_2). For the controlled experiment, we purged pure Ar into the electrolyte at the beginning of the electrochemical reduction reaction. After that, Ar gas was replaced by CO_2 to test the ECR performance. **b** CO FE of the normal experiment and controlled experiment. The potential was -0.8 V vs. RHE.

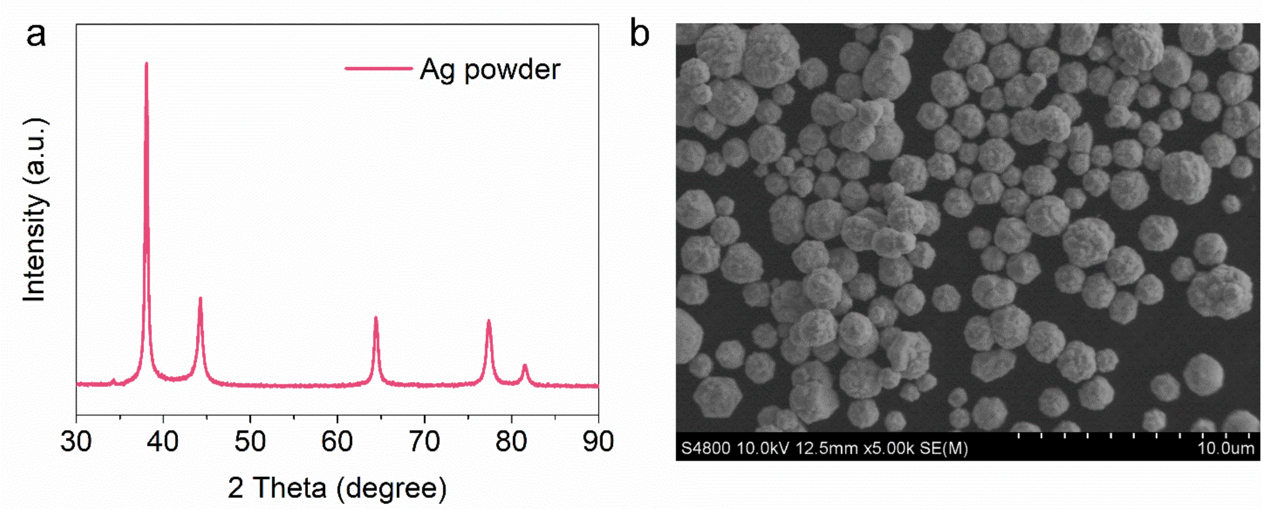

Supplementary Figure 12. Characterizations of the Ag powder. **a** XRD pattern. **b** SEM image.

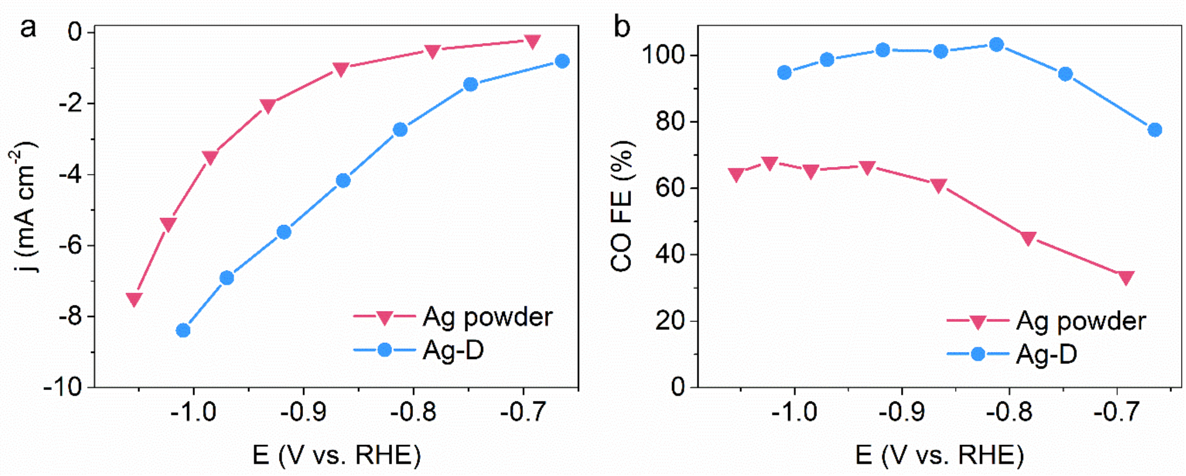

Supplementary Figure 13. ECR performance measured in an H-cell reactor. **a** Total current density (j) curves of Ag-D and Ag powder catalysts. **b** FE of CO for Ag-D and Ag powder catalysts.

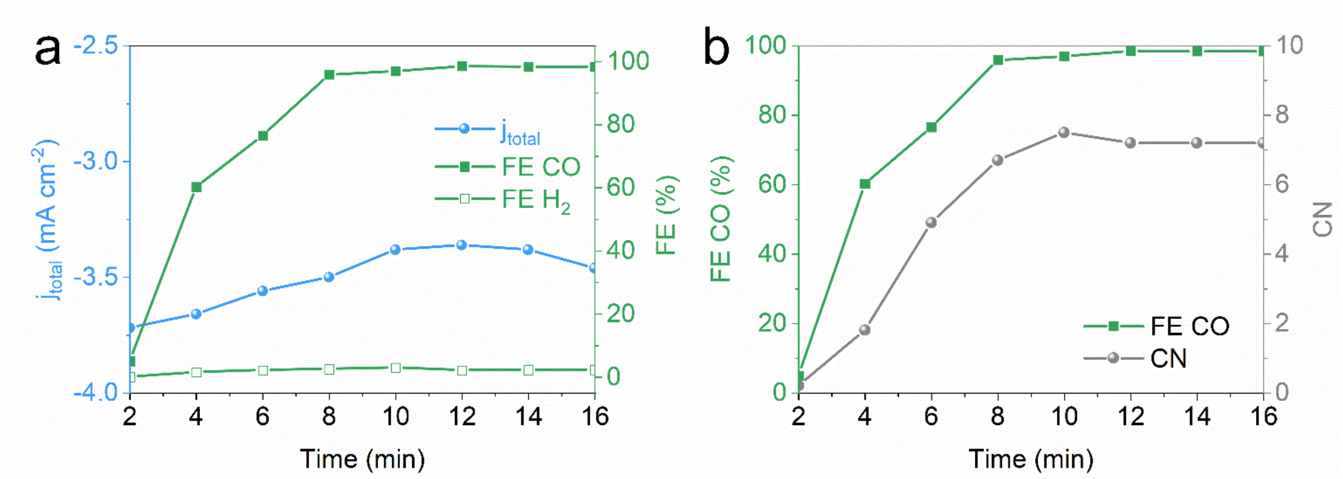

Supplementary Figure 14. Correlation between the ECR performance and the fast XAS result. **a** Time-resolved ECR performance measured in an H-cell reactor. **b** FE of CO and CN plots as a function of time.

To achieve the required time resolution corresponding to the timescale of fast XAS, the volume of headspace left in the cathodic room was reduced to ~10 mL by adding more electrolyte. The flow rate of CO_2 fed into the cathodic electrolyte was also increased to 40 mL/min. These modifications could ensure a shorter buffer time for gas products in the cathodic headspace.

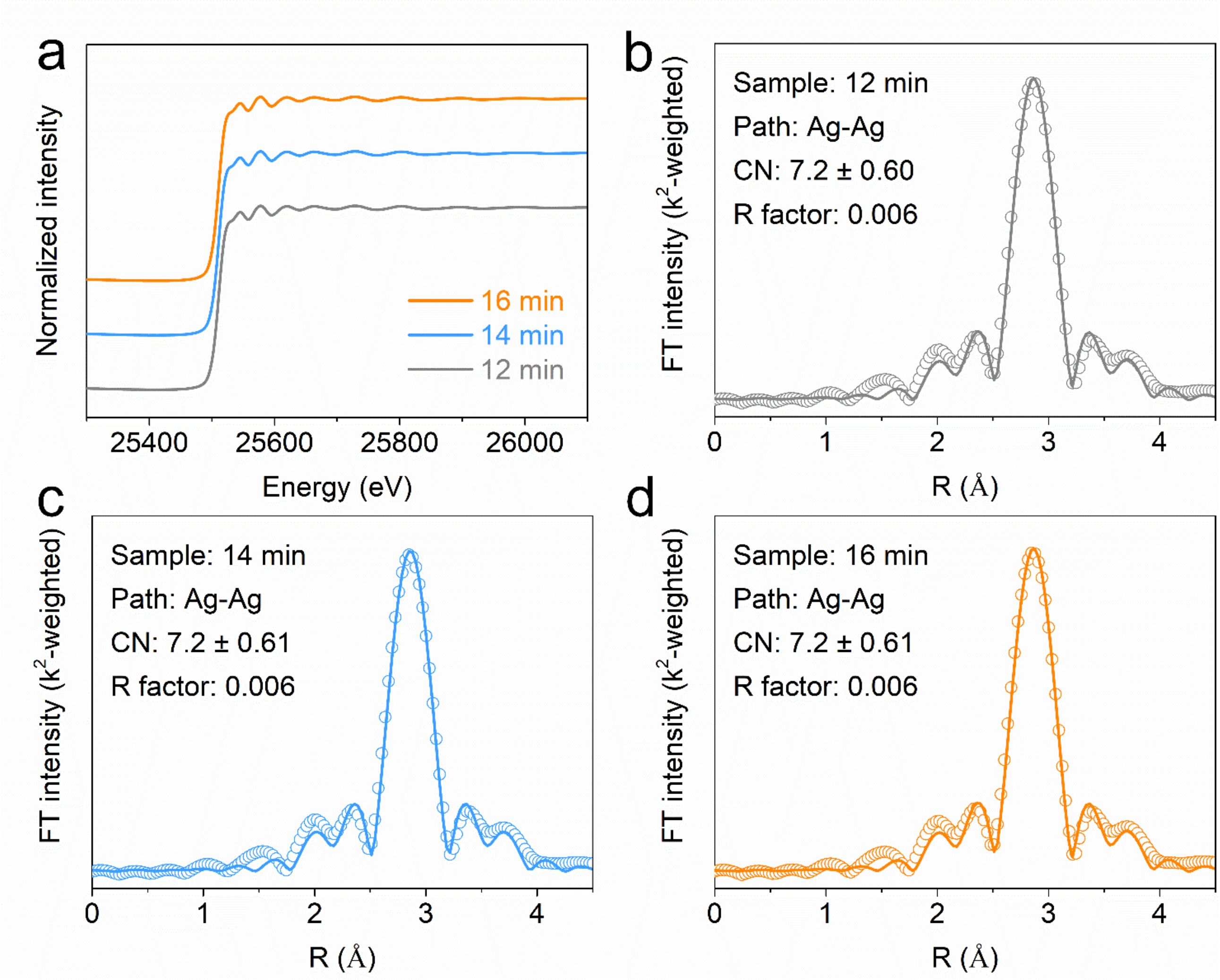

Supplementary Figure 15. Fast operando XAS results. **a** Normalized XANES profiles. The k^2-weighted FT EXAFS spectra (circles) and the fitting result (lines) for the Ag-D catalyst collected at **b** ~12 min, **c** ~14 min, and **d** ~16 min.

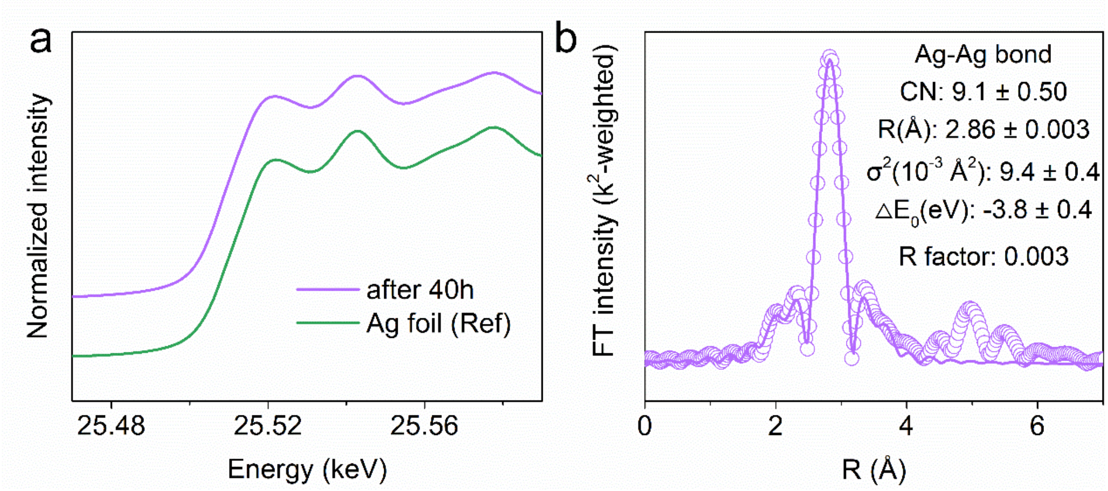

Supplementary Figure 16. Ex-situ XAFS measurements of Ag-D catalyst after a 40-h reaction. **a** Normalized XANES profiles at Ag K-edge. **b** k^2-weighted FT EXAFS spectrum (circles) and the fitting result (line).

Supplementary Table 2. Summary of ECR performance in $KHCO_3$ electrolyte for various representative Ag based catalysts.

Catalysts	Electrolyte	j_{CO} (mA cm^{-2})	E (V vs. RHE)	CO FE (%)	Stability (h)	Ref.
**Ag-D**	**0.1 M $KHCO_3$**	**2.73**	**-0.81**	**~100**	**120**	**This work**
Ag-200 nm NWA	0.5 M $KHCO_3$	4.47	-0.6	91	24	1
Ag-30 nm NWA	0.5 M $KHCO_3$	4.01	-0.6	59	24	1
np-Ag	0.5 M $KHCO_3$	8	-0.5	90	8	2
OD-Ag	0.1 M $KHCO_3$	1.15	-0.8	89	2	3
Polycrystalline Ag foil	0.1 M $KHCO_3$	0.13	-0.8	20	2	3
Tri-Ag-NPs	0.1 M $KHCO_3$	~1.25	-0.856	~96	168	4
porous Ag	0.5 M $KHCO_3$	~12.8	-1.7 *	~90	4	5
disordered Ag	0.1 M $KHCO_3$	~1.6	-0.7	~80	5	6
3 nm Ag	0.1 M $KHCO_3$	~0.8	N/A **	~40	5	6
5 nm Ag	0.1 M $KHCO_3$	~1.6	N/A	~80	5	6
11 nm Ag	0.1 M $KHCO_3$	~1.2	N/A	~60	5	6

*: This value of potential (-1.7 V) employed a saturated calomel electrode (SCE) as the reference electrode.

**: the data was unavailable.

Supplementary Table 3. Summary of η, FE, and EE for various catalysts in $KHCO_3$ electrolyte.

Catalysts	Electrolyte	η (V)	FE (%)	EE (%)	Ref.
Ag-D	0.1 M $KHCO_3$	0.7	~100	65.7	**This work**
Ag-200 nm NWA	0.5 M $KHCO_3$	0.49	91	66.6	1
np-Ag	0.5 M $KHCO_3$	0.49	92	67.4	2
AgP_2	0.5 M $KHCO_3$	0.69	82	54.1	7
Ag nanowires	0.5 M $KHCO_3$	0.79	80	50.3	8
$Ag_{15}Pd_{85}$	0.1 M $KHCO_3$	0.69	~100	66.0	9
Tri-Ag-NPs	0.1 M $KHCO_3$	0.746	~96	61.7	4
OD-Ag	0.1 M $KHCO_3$	0.69	89	58.7	3
disordered Ag	0.1 M $KHCO_3$	0.59	~80	55.5	6
L25-Ag-NCs	0.1 M $KHCO_3$	0.746	99	63.6	10
SPC-Ag	0.1 M $KHCO_3$	0.79	93	58.5	11
3D porous Ag	0.1 M $KHCO_3$	0.92	96	56.9	12
Au nanowires	0.5 M $KHCO_3$	0.24	94	79.7	13
Au nanoneedles	0.5 M $KHCO_3$	0.24	95	80.6	14
core shell Cu/SnO_2	0.5 M $KHCO_3$	0.59	93	64.6	15
A-Ni-NSG	0.5 M $KHCO_3$	0.39	97	75.1	16
Fe^{3+}-N-C	0.5 M KHCO3	0.34	91	72.6	17
atomic Ir	0.5 M KHCO3	0.55	97.6	69.2	18

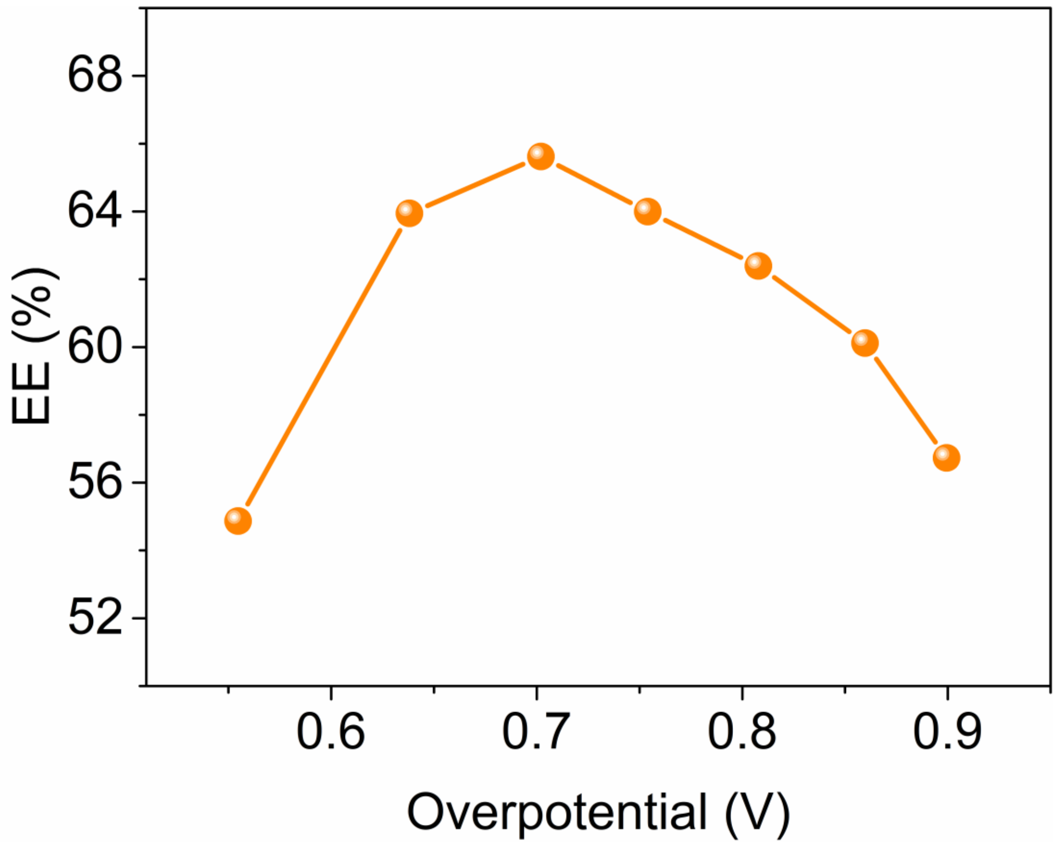

Supplementary Figure 17. EE of Ag-D at different overpotentials.

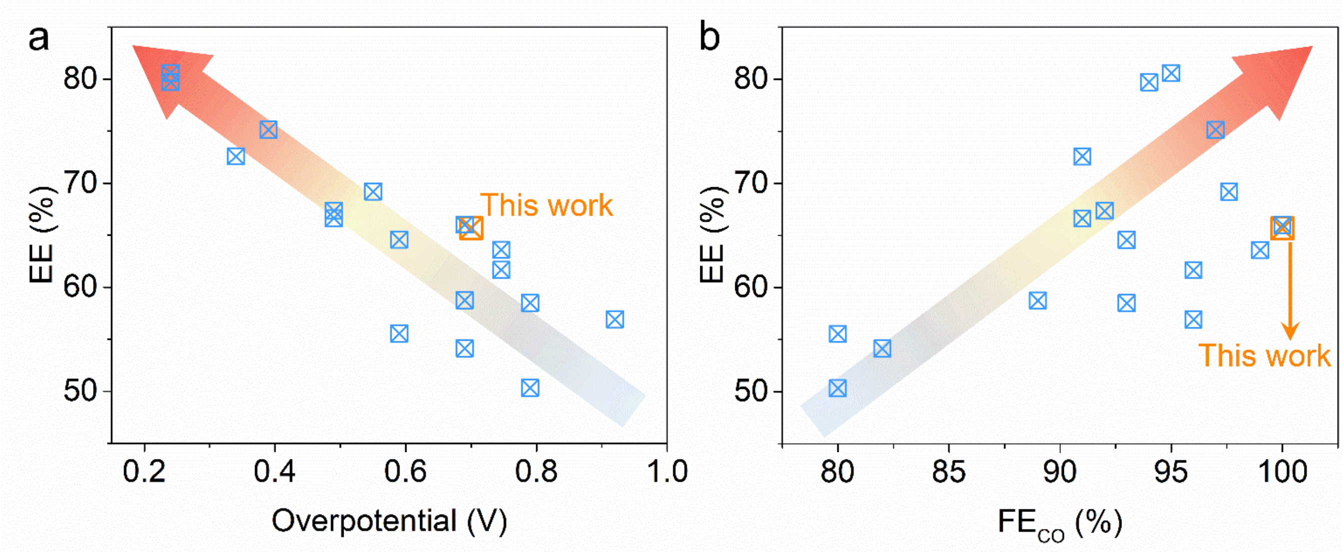

Supplementary Figure 18. Comparison on EE as the function of **a** Overpotential, and **b** FE of CO. The blue squares indicate the catalysts collected from literatures.

Supplementary note 1. The Adsorption Energy Calculation

The adsorption energy was calculated as[19]:

$$\Delta E_{\mathrm{ads}} = E_{\mathrm{ads/slab}} - E_{\mathrm{slab}} - E_{\mathrm{ads}} \qquad \text{(eq. S1)}$$

where $E_{\mathrm{ads/slab}}$, E_{slab} and E_{ads} are the energy of the adsorbate on the surface, the energy of the clean surface and the energy of the isolated adsorbate, respectively. The negative ΔE_{ads} indicates exothermic adsorption. The more negative of ΔE_{ads}, the more stable is the adsorption.

Supplementary note 2. The Free Energy Calculation

The free energy of each state is calculated through eq. S2:

$$G = E + ZPE + \int C_p dT - TS \quad \text{(eq. S2)}$$

Where E is the electronic energy obtained directly from DFT calculation. The zero point energy (ZPE), enthalpic correction ($\int C_p dT$) and entropic correction (TS) were calculated based on the vibrational analysis in the harmonic oscillator approximation and were used to convert the electronic energy into the free energy at 298.15 K. Frequencies of all 3N degrees of freedom of the adsorbate were treated as vibrational, and any changes of the vibrations of the substrate surface were assumed to be minimal to be neglected, which are in accordance with previous publications (see **Supplementary Table 3** for the calculated thermodynamic properties)[20]. The standard state pressure of 101,325 Pa was applied for gaseous species (CO_2, H_2); a fugacity of 3534 Pa was used for H_2O (l), corresponding to the vapor pressure of water at 300 K (Ref[21]).

The free energy of gaseous CO was obtained through the following reaction at standard state:

$$CO_2(g)+H_2(g) \leftrightarrow CO(g)+H_2O(l) \quad \text{(re. S1)}$$

This is because the energy of CO obtained using the pseudopotential method lead to un-negligible error of the reaction free energy (ΔG_r) of re. S1 with respect to the experimental value (ΔG_r =0.24 eV). Then we have:

$$G(CO(g))= \Delta G_r - G(H_2O(l))+ G(CO_2(g))+ G(H_2(g)) \quad \text{(eq. S3)}$$

The computational hydrogen electrode (CHE) model was used to include the electrode potential correction and the pH correction to the free energy of each state[21]. In this model, the free energy of a proton-electron pair at U *vs* RHE is defined to be:

$$G(H^+ + e^-) = 1/2G(H_2) - 2.303RT \times pH - e_0U \quad \text{(eq. S4)}$$

where e_0 is the elementary positive charge of an electron and R is the gas constant. For electrochemical ECR to CO, there are four elementary states as follows:

$CO_2(g) + 2H^+ + 2e^- + *$ (1)

$*COOH + H^+ + e^-$ (2)

$*CO + H_2O(l)$ (3)

$CO(g) + H_2O(l) + *$ (4)

so

$$G(1) = G(CO_2(g)) + 2(1/2G(H_2) - 2.303RT*pH - e_0U) + G(surf) \quad \text{(eq. S5)}$$

$$G(2) = G(*COOH+surf) + 1/2G(H_2) - 2.303RT*pH - e_0U \quad \text{(eq. S6)}$$

$G(3) = G(\text{*CO+surf}) + G(H_2O(l))$ (eq. S7)

$G(4) = G(CO(g)) + G(H_2O(l)) + G(\text{surf})$ (eq. S8)

Based on eqs. S5-S8, we can obtain the free energy profile which provides the thermodynamical information of the reaction.

The limiting potential (U_L) is defined as the potential at which the electrochemical elementary step becomes exergonic (or downhill in free energy). It can be calculated as:

$$U_L = -\frac{\Delta G_r|0\ V}{e} \qquad \text{(eq. S9)}$$

The difference between the equilibrium potential of the overall electrochemical reduction of CO_2 to CO and the min$\{U_{L_1}, U_{L_2}\dots U_{L_M}\}$ (M is the number of the electrochemical elementary steps) is the overpotential[22].

Supplementary note 3. DFT calculations on the Ag (100), and (110) related facets.

The Ag (111), (100) and (110) facets were corresponding to the three intensive peaks observed in the XRD pattern (**Fig. 1a**), and are often considered as the representative models in theoretical studies[23]. Based on the result on Ag (111), we constructed proper vacancy models for Ag (100) and Ag (110). The most stable binding geometry of COOH on pristine Ag (100) (ΔE_{ads} = -1.98 eV) is similar to that on pristine Ag (111) (ΔE_{ads} = -2.0 eV) (**Fig. 5**, and **Supplementary Fig. 22**). The tiny tilt of COOH towards the vacancy center on defect Ag (100) slightly shortens the O-Ag atomic distances (carbonyl(O)-Ag distance: 3.25 Å, hydroxyl(O)-Ag distance: 3.20 Å) compared with the case on pristine surface (carbonyl(O)-Ag distance: 3.46 Å, hydroxyl(O)-Ag distance: 3.33 Å), which results in slightly stronger binding of COOH with ΔE_{ads} = -2.04 eV. On Ag (110)-pristine surface, the O-C-O plane of COOH is almost perpendicular to the metal surface with the C atom and the carbonyl O atom interact with two Ag atoms separately (C-Ag distance: 2.15 Å, carbonyl(O)-Ag distance: 2.42 Å), resulting in a ΔE_{ads} of -2.17 eV. On Ag(110)-vacancy surface, COOH stably binds at the vacancy edge site (**Supplementary Fig. 23**), in which the C atom and the two O atoms interact with three Ag atoms separately (C-Ag distance: 2.18 Å, carbonyl(O)-Ag distance: 2.42 Å, hydroxyl(O)-Ag distance: 2.57 Å) and the O-C-O plane tilts toward the vacancy central area. This geometry leads to a ΔE_{ads} of -2.26 eV. The binding geometry of CO at the vacancy edge site on Ag(100)/Ag(110)-vacancy surface is similar to that on Ag(100)/Ag(110)-pristine surface, which is stabilized by the C-Ag interaction. The vacancy results in slightly stronger binding of CO.

The computed reaction free energy diagrams (**Supplementary Fig. 24**) depict that on all three studied Ag crystal surfaces, the activation of carbon dioxide by protonation to form *COOH is the potential determining step. The Ag (110) surface has the highest limiting potentials (U_L) among three surfaces, revealing that Ag (110) has the highest CO evolution activity, which is consistent with the previous study[23]. The strengthened binding of COOH results in higher limiting potentials on the vacancy containing surfaces than that on the pristine surfaces.

Supplementary Table 4. The optimized lattice parameters for Ag (111) slabs with and without vacancies, respectively.

models	***a*** **(Å)**	***b*** **(Å)**	***c*** **(Å)**	α	β	γ
p(3×3)-pristine	14.95	14.95	24.70	90°	90°	120°
Ag (111)-4% vacancy	14.77	14.77	24.79	90°	90°	120°
p(2×2)-pristine	9.96	9.96	24.70	90°	90°	120°
Ag (111)-8% vacancy	10.01	10.01	24.73	90°	90°	120°
Ag (111)-17% vacancy	9.96	10.03	24.78	89.71°	90.16°	120.24°

Supplementary Table 5. Computed thermodynamic properties in eV.

	**ZPE**	$\int C_p dT$	**-TS**
CO_2(g)	0.31	0.09	-0.66
H_2(g)	0.27	0.08	-0.40
H_2O(l)	0.57	0.10	-0.68
*COOH@Ag(111)-pristine	0.59	0.02	-0.16
*CO@ Ag(111)-pristine	0.16	0.09	-0.19
*COOH@Ag(111)-4% vacancy	0.59	0.01	-0.14
*CO@Ag(111)-4% vacancy	0.17	0.08	-0.18
*COOH@Ag(111)-8% vacancy	0.58	0.09	-0.20
*CO@Ag(111)-8% vacancy	0.16	0.07	-0.15
*COOH@Ag(111)-17% vacancy	0.57	0.09	-0.20
*CO@Ag(111)-17% vacancy	0.16	0.07	-0.16

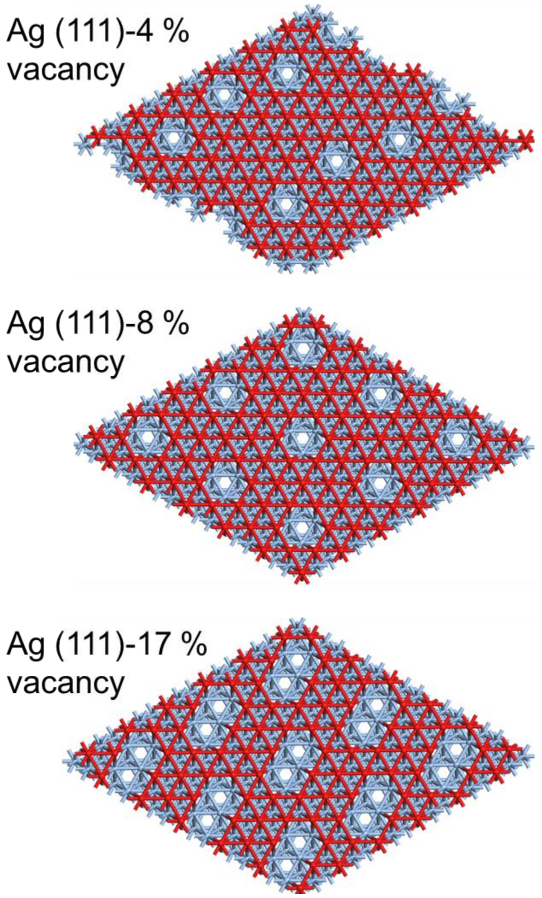

Supplementary Figure 19. The structures of vacancy models. The Ag atoms of the first atomic layers are labeled in red.

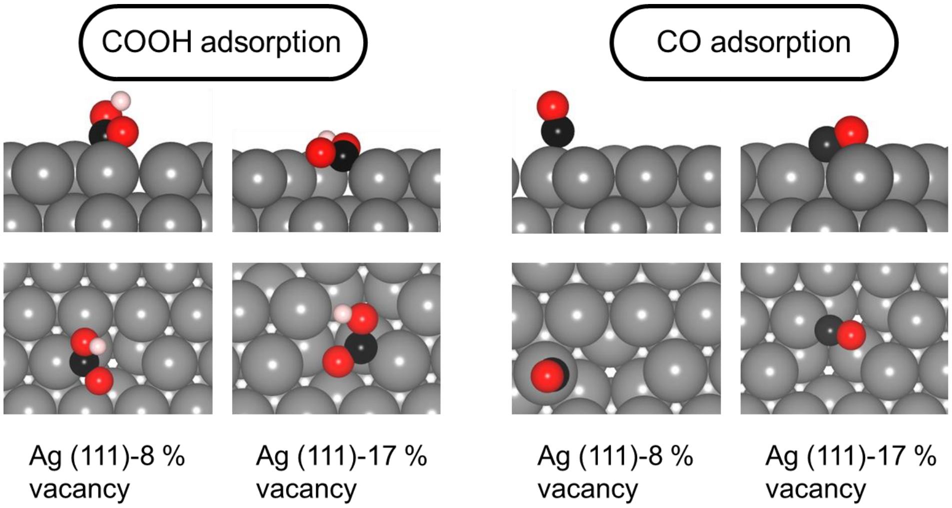

Supplementary Figure 20. The most preferred binding geometries of adsorbate on pristine Ag (111) and vacancy-defected Ag (111).

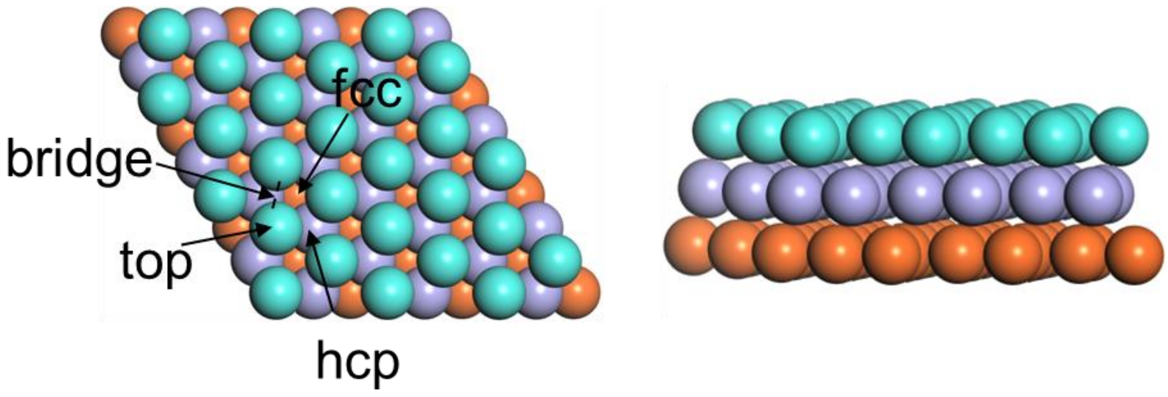

Supplementary Figure 21. The three layer Ag (111) model. The four high symmetry adsorption sites (top site, fcc hollow site, hcp hollow site and bridge site) were denoted by arrows.

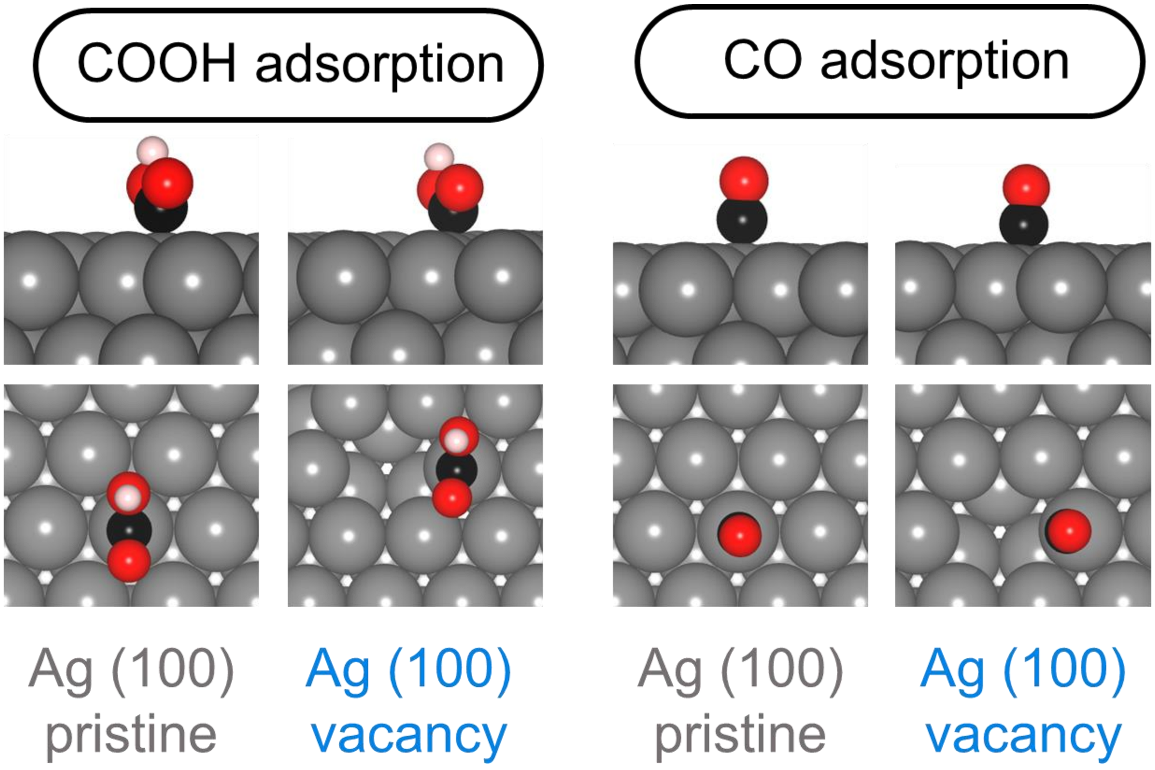

Supplementary Figure 22. The most preferred binding geometries of adsorbates on pristine Ag (100) and vacancy-defected Ag (100). Side view: above; top view: below. Colour codes: Ag, grey; C, black; O, red; H, pink.

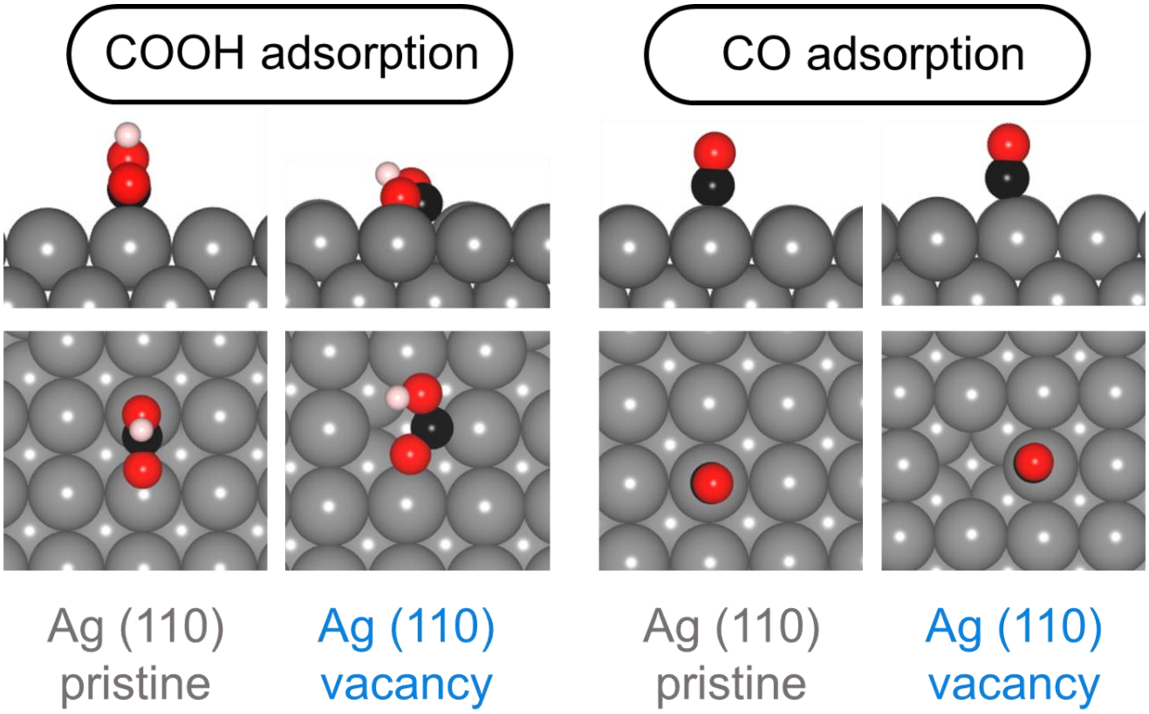

Supplementary Figure 23. The most preferred binding geometries of adsorbates on pristine Ag (110) and vacancy-defected Ag (110). Side view: above; top view: below. Colour codes: Ag, grey; C, black; O, red; H, pink.

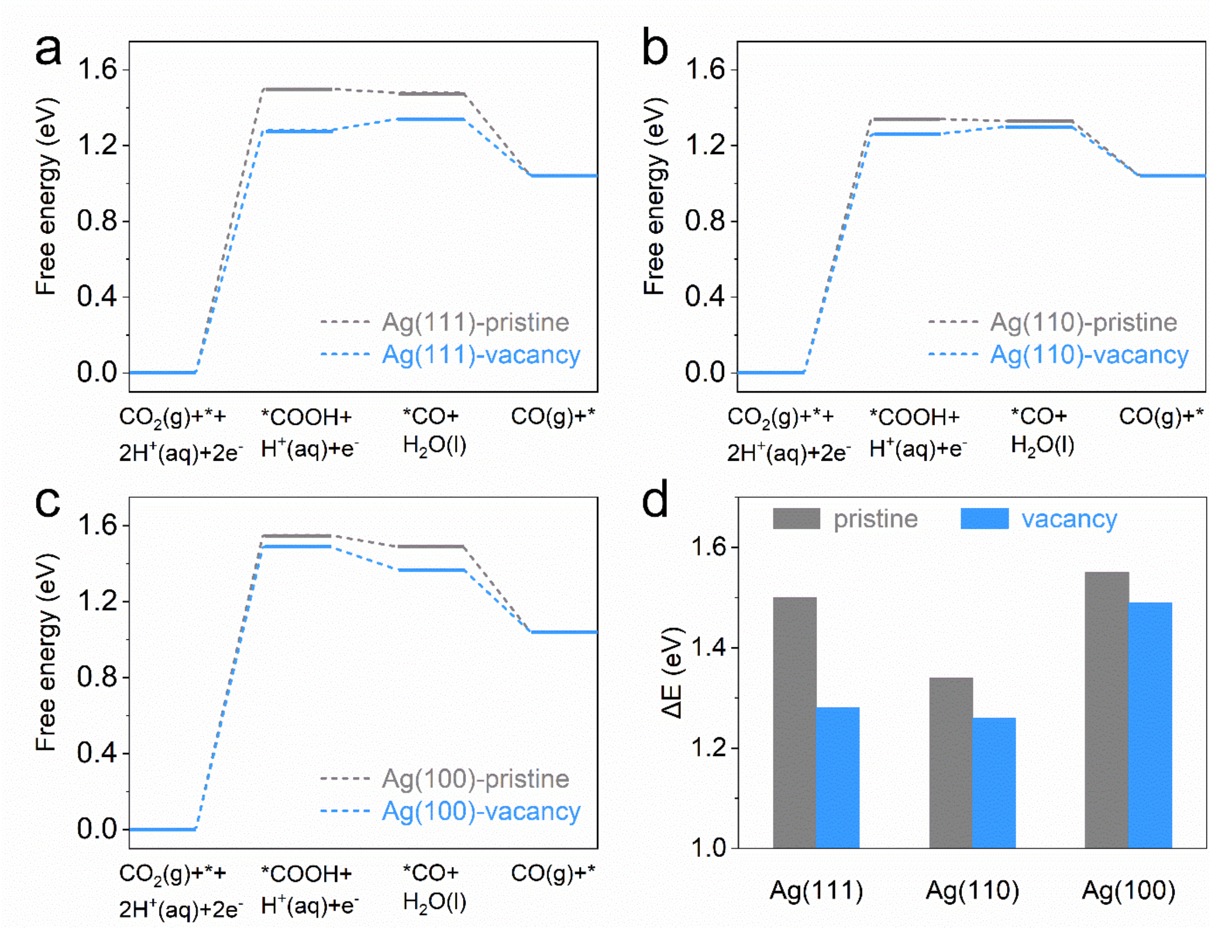

Supplementary Figure 24. Theoretical study on ECR performance of various Ag facets. Free energy diagrams on **a** Ag (111)-pristine and Ag (111)-vacancy surface, **b** Ag (110)-pristine and Ag (111)-vacancy surface, and **c** Ag (100)-pristine and Ag (100)-vacancy surface. **d** The change of free energy for the first step of ECR to produce *COOH on Ag (111), (110), and (100). Grey and blue lines represent pristine and vacancy surface, respectively.

Supplementary references

1. Luan, C. et al. High-performance carbon dioxide electrocatalytic reduction by easily fabricated large-scale silver nanowire arrays. *ACS Appl. Mater. Interfaces* **10**, 17950-17956 (2018).

2. Lu, Q. et al. A selective and efficient electrocatalyst for carbon dioxide reduction. *Nat. Commun.* **5**, 3242 (2014).

3. Ma, M., Trześniewski, B. J., Xie, J. & Smith, W. A. Selective and efficient reduction of carbon dioxide to carbon monoxide on oxide-derived nanostructured silver electrocatalysts. *Angew. Chem. Int. Ed.* **55**, 9748-9752 (2016).

4. Liu, S. et al. Shape-dependent electrocatalytic reduction of CO_2 to CO on triangular silver nanoplates. *J. Am. Chem. Soc.* **139**, 2160-2163 (2017).

5. Wang, H. et al. Enhanced CO selectivity and stability for electrocatalytic reduction of CO_2 on electrodeposited nanostructured porous Ag electrode. *J. CO_2 Util.* **15**, 41-49 (2016).

6. Deng, W., Zhang, L., Dong, H., Chang, X., Wang, T. & Gong, J. Achieving convenient CO_2 electroreduction and photovoltage in tandem using potential-insensitive disordered Ag nanoparticles. *Chem. Sci.* **9**, 6599-6604 (2018).

7. Li, H. et al. Colloidal silver diphosphide (AgP_2) nanocrystals as low overpotential catalysts for CO_2 reduction to tunable syngas. *Nat. Commun.* **10**, 5724 (2019).

8. Xi, W., Ma, R., Wang, H., Gao, Z., Zhang, W. & Zhao, Y. Ultrathin Ag nanowires electrode for electrochemical syngas production from carbon dioxide. *ACS Sustain. Chem. Eng.* **6**, 7687-7694 (2018).

9. Cui, M. et al. AgPd nanoparticles for electrocatalytic CO_2 reduction: bimetallic composition-dependent ligand and ensemble effects. *Nanoscale* **12**, 14068-14075 (2020).

10. Liu, S., Sun, C., Xiao, J. & Luo, J.-L. Unraveling structure sensitivity in CO_2 electroreduction to near-unity CO on silver nanocubes. *ACS Catal.* **10**, 3158-3163 (2020).

11. Fan, T. et al. Electrochemically driven formation of sponge-like porous silver nanocubes toward efficient CO_2 electroreduction to CO. *ChemSusChem* **13**, 2677-2683 (2020).

12. Abeyweera, S. C., Yu, J., Perdew, J. P., Yan, Q. & Sun, Y. Hierarchically 3D porous Ag nanostructures derived from silver benzenethiolate nanoboxes: enabling CO_2 reduction with a near-unity selectivity and mass-specific current density over 500 A/g. *Nano letters* **20**, 2806-2811 (2020).

13. Zhu, W. et al. Active and selective conversion of CO_2 to CO on ultrathin Au nanowires. *J. Am. Chem. Soc.* **136**, 16132-16135 (2014).

14. Liu, M. et al. Enhanced electrocatalytic CO_2 reduction via field-induced reagent concentration. *Nature* **537**, 382-386 (2016).

15. Li, Q. et al. Tuning Sn-catalysis for electrochemical reduction of CO_2 to CO via the core/shell Cu/SnO_2 structure. *J. Am. Chem. Soc.* **139**, 4290-4293 (2017).

16. Yang, H. B. et al. Atomically dispersed Ni(I) as the active site for electrochemical CO_2 reduction. *Nat. Energy* **3**, 140-147 (2018).

17. Gu, J., Hsu, C.-S., Bai, L., Chen, H. M. & Hu, X. Atomically dispersed Fe^{3+} sites catalyze efficient CO_2 electroreduction to CO. *Science* **364**, 1091 (2019).

18. Sun, X. et al. Aqueous CO_2 reduction with high efficiency using α-Co(OH)$_2$-supported atomic Ir electrocatalysts. *Angew. Chem. Int. Ed.* **58**, 4669-4673 (2019).

19. Chen, B. W. J., Kirvassilis, D., Bai, Y. & Mavrikakis, M. Atomic and molecular adsorption on Ag(111). *J. Phys. Chem. C* **123**, 7551-7566 (2019).

20. Jiao, Y., Zheng, Y., Chen, P., Jaroniec, M. & Qiao, S.-Z. Molecular scaffolding strategy with synergistic active centers to facilitate electrocatalytic CO_2 reduction to hydrocarbon/alcohol. *J. Am. Chem. Soc.* **139**, 18093-18100 (2017).

21. Nørskov, J. K. et al. Origin of the overpotential for oxygen reduction at a fuel-cell cathode. *J. Phys. Chem. B* **108**, 17886-17892 (2004).

22. Peterson, A. A. & Nørskov, J. K. Activity descriptors for CO_2 electroreduction to methane on transition-metal catalysts. *J. Phys. Chem. Lett.* **3**, 251-258 (2012).

23. Clark, E. L. et al. Influence of atomic surface structure on the activity of Ag for the electrochemical reduction of CO_2 to CO. *ACS Catal.* **9**, 4006-4014 (2019).
